# Walk on the Wild Side: Estimating the Global Magnitude of Visits to Protected Areas

**DOI:** 10.1371/journal.pbio.1002074

**Published:** 2015-02-24

**Authors:** Andrew Balmford, Jonathan M. H. Green, Michael Anderson, James Beresford, Charles Huang, Robin Naidoo, Matt Walpole, Andrea Manica

**Affiliations:** 1 Department of Zoology, University of Cambridge, Cambridge, United Kingdom; 2 Woodrow Wilson School of Public and International Affairs, Princeton University, Princeton, New Jersey, United States of America; 3 World Wildlife Fund, Washington, D.C., United States of America; 4 Cambridge Institute for Sustainability Leadership, Cambridge, United Kingdom; 5 UNEP–World Conservation Monitoring Centre, Cambridge, United Kingdom

## Abstract

How often do people visit the world’s protected areas (PAs)? Despite PAs covering one-eighth of the land and being a major focus of nature-based recreation and tourism, we don’t know. To address this, we compiled a globally-representative database of visits to PAs and built region-specific models predicting visit rates from PA size, local population size, remoteness, natural attractiveness, and national income. Applying these models to all but the very smallest of the world’s terrestrial PAs suggests that together they receive roughly 8 billion (8 x 109) visits/y—of which more than 80% are in Europe and North America. Linking our region-specific visit estimates to valuation studies indicates that these visits generate approximately US $600 billion/y in direct in-country expenditure and US $250 billion/y in consumer surplus. These figures dwarf current, typically inadequate spending on conserving PAs. Thus, even without considering the many other ecosystem services that PAs provide to people, our findings underscore calls for greatly increased investment in their conservation.

## Abstract 2

A new global analysis indicates that between them, the world’s protected areas receive approximately 8 billion visits each year, with an economic value of hundreds of billions of dollars annually.

Enjoyment of nature, much of it in protected areas (PAs), is recognised as the most prominent cultural ecosystem service [1–3], yet we still lack even a rough understanding of its global magnitude and economic significance. Large-scale assessments have been restricted to regional or biome-specific investigations [4–8] (but see [9]). There are good reasons for this. Information on visit rates is limited, widely scattered, and confounded by variation in methods [10,11]. Likewise, estimates of the value of visits vary greatly—geographically, among methods, and depending on the component of value being measured [12–14]. Until now, these problems have prevented data-driven analysis of the worldwide scale of nature-based recreation and tourism. But with almost all the world’s governments committed (through the Aichi Biodiversity Targets [15]) to integrating biodiversity into national accounts, policymakers require such gaps in our knowledge of natural capital to be filled.

We tackled this shortfall in our understanding of a major ecosystem service by focusing on terrestrial PAs, which cover one-eighth of the land [16] and are a major focus of nature-based recreation and tourism. We compiled data on visit rates to over 500 PAs and built region-specific models, which predicted variation in visitation in relation to the properties of PAs and to local socioeconomic conditions. Next, we used these models to estimate visit rates to all but the smallest of the world’s terrestrial PAs. Last, by summing these estimates by region and combining the totals with region-specific medians for the value of nature visits obtained from the literature, we derived approximate estimates of the global extent and economic significance of PA visitation.

Given the scarcity of data on visits to PAs, our approach was to use all available information (although we excluded marine and Antarctic sites, and International Union for Conservation of Nature (IUCN) Category I PAs where tourism is typically discouraged; for further details of data collection and analysis see Materials and Methods). This generated a database of visitor records for 556 PAs spread across 51 countries and included 2,663 records of annual visit numbers over our best-sampled ten-year period (1998–2007) (S1 Table). Mean annual visit rates for individual PAs in this sample ranged from zero to over 10 million visits/y, with a median across all sampled PAs of 20,333 visits/y.

We explored this variation by modelling it in relation to a series of biophysical and socioeconomic variables that might plausibly predict visit rates (after refs [6,7,17]): PA size, local population size, PA remoteness, a simple measure of the attractiveness of the PA’s natural features, and national income (see Materials and Methods for a priori predictions). For each of five major regions, we performed univariate regressions (S2 Table) and then built generalised linear models (GLMs) in an effort to predict variation in observed visit rates. While the GLMs had modest explanatory power within regions (S3 Table), together they accounted for 52.9% of observed global variation in visit rates. Associations with individual GLM variables—controlling for the effects of other variables—differed regionally in their strength but broadly matched our predictions (S1 Fig.). Visit rates increased with local population size (in Europe), decreased with remoteness (everywhere apart from Asia/Australasia), increased with natural attractiveness (in North and Latin America), and increased with national income (everywhere else). Controlling for these variables, visit rates were highest in North America, lower in Asia/Australasia and Europe, and lowest in Africa and Latin America.

To quantify how often people visit PAs as a whole, we used our region-specific GLMs to estimate visit rates to 94,238 sites listed in the World Database on Protected Areas (WDPA) [18]). We again excluded marine, Antarctic, and Category I PAs, as well as almost 40,000 extremely small sites which were below the size (10 ha) of the smallest PA in our sample (S2 Fig.). The limited power of our GLMs and significant errors in the WDPA mean our estimates of visit rates should be treated with caution for individual sites or (when aggregated to national level) for smaller countries. However, the larger-scale patterns they reveal are marked. Estimated median visit rates per PA (averaged within countries) are lowest in Africa (at around 3,000/y) and Latin America (4,000/y), and greatest in North America (350,000/y) (S3 Table). When visit rates are aggregated across all PAs within a country, pronounced regional differences in the numbers of PAs (with relatively few in Africa and Latin America) magnify these patterns and indicate that while many African countries have <100,000 PA visits/y, PAs in the United States receive a combined total of over 3 billion visits/y (Fig. 1). This variation is underscored when aggregate PA visit rates are standardised by the annual number of non-workdays and total population size of each region: across Europe we reckon there are ~5 PA visits/100 non-work person-days; for North America, the figure is ~10 visits/100 non-work person-days respectively, while for each other region our estimates are <0.3 visits/100 non-work person-days.

**Fig 1:**
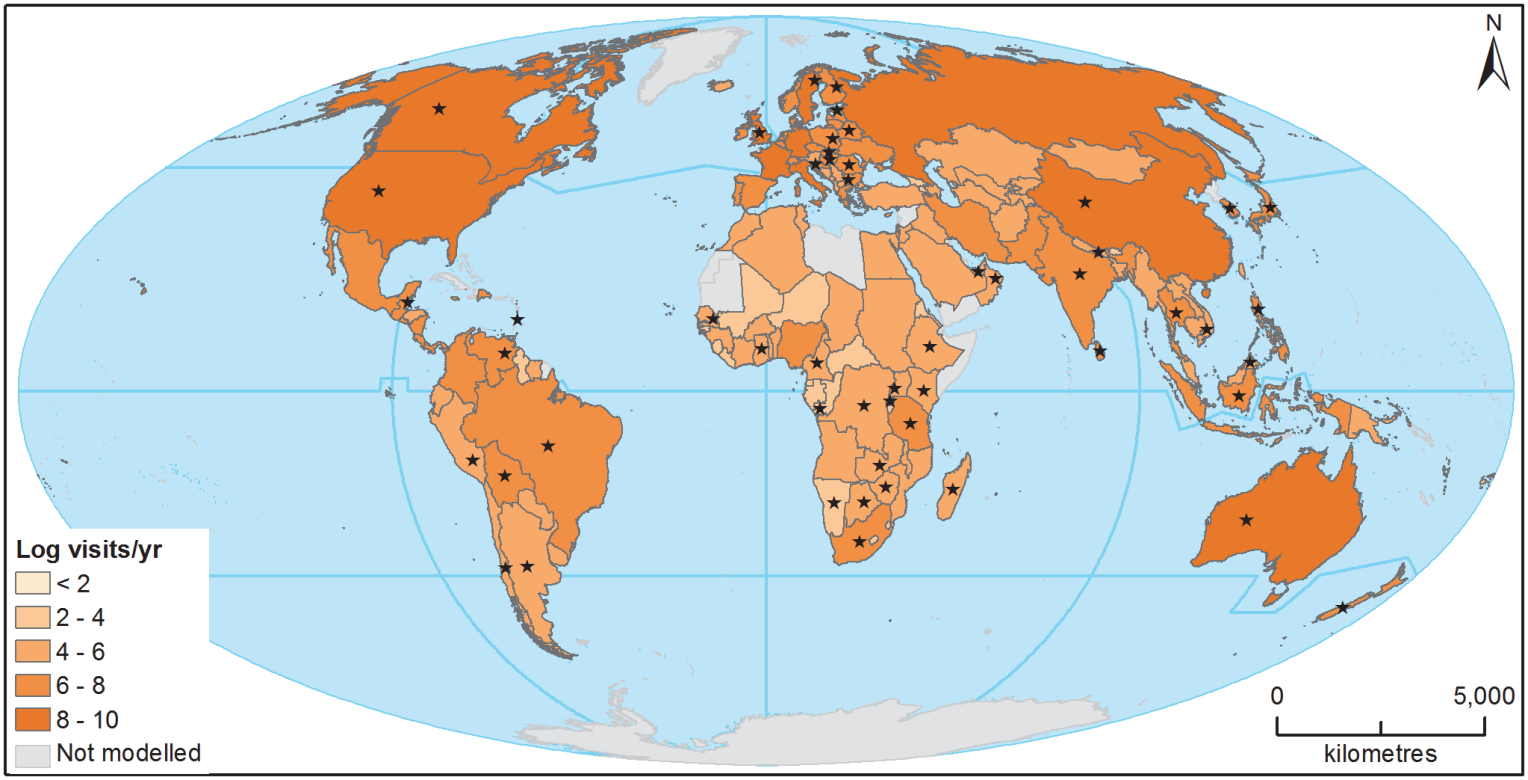
Estimated total PA visit rates for each country. Totals (which are log_10_-transformed) were derived by applying the relevant regional GLM (S3 Table) to all of a country’s terrestrial PAs (excluding those <10 ha, and marine and IUCN Category I PAs) listed in the WDPA [18]. Asterisks show countries for which we had visit rate observations.

Summing our aggregate estimates of PA visits suggests that between them, the world’s terrestrial PAs receive approximately 8 billion visits/y. Of these, we estimate 3.8 billion visits/y are in Europe (where more than half of the PAs in the WDPA are located) and 3.3 billion visits/y are in North America (S3 Table). These numbers are strikingly large. However, given our confidence intervals (95% CIs for the global total: 5.4–18.5 billion/y) and considering several conservative aspects of our calculations (e.g., the exclusion of ~40,000 very small sites and the incomplete nature of the WDPA), we consider it implausible that there are fewer than 5 billion PA visits worldwide each year. Three national estimates support this view: 2.5 billion visitdays/y to US PAs in 1996 [4], >1 billion visits/y (albeit many of them cultural rather than nature-based) to China’s National Parks in 2006 [19], and 3.2–3.9 billion visits/y to all British “ecosystems” (most of which are not in PAs) in 2010 [7].

Finally, what can be inferred about the economic significance of visits on this scale? Economists working on tourism distinguish two main, non-overlapping components of value [12]: direct expenditure by visitors (an element of economic impact, calculated from spending on fees, travel, accommodation, etc.); and consumer surplus (a measure of economic value which arises because many visitors would be prepared to pay more for their visit than they actually have to, and which is defined as the difference between what visitors would be prepared to pay for a visit and what they actually spend; consumer surplus is typically quantified using travel cost or contingent valuation methods). We conducted an extensive literature search to derive median (but conservative) figures for each type of value for each region (S4 Table). Applying these to our corresponding estimates of visit rates and summing across regions yields an estimate of global gross direct expenditure associated with PA visits (within-country only, and excluding indirect and induced expenditure) of ~US $600 billion/y worldwide (at 2014 prices). The corresponding figure for global consumer surplus is ~US $250 billion/y.

Such numbers are unavoidably imprecise. Uncertainty in our modelled visit rates and the wide variation in published estimates of expenditure and consumer surplus mean that they could be out by a factor of two or more. However, comparison with calculations that visits to North American PAs alone have an economic impact of $350–550 billion/y [4] and that direct expenditure on all travel and tourism worldwide runs at $2,000 billion/y [20] suggests our figures are of the correct order of magnitude, and that the value of PA visitation runs into hundreds of billions of dollars annually.

These results quantify, we believe for the first time, the scale of visits to the world’s PAs and their approximate economic significance. We currently spend <$10 billion/y in safeguarding PAs [21]—a figure which is widely regarded as grossly insufficient [21–25]. Even without considering the many other benefits which PAs provide [22], our estimates of the economic impact and value of PA visitation dwarf current expenditure—highlighting the risks of underinvestment in conservation, and suggesting substantially increased investments in protected area maintenance and expansion would yield substantial returns.

## Supporting Information

S1 Fig Visit rates plotted against each predictor variable. Plots show observed visit rates (adjusted for every other predictor variable) against each predictor variable (top to bottom) for each region (left to right). Values for mean visit rate, PA size, local population size, remoteness, and national income are all log_10_-transformed (after adding one to all values of mean visit rate and local population size and remoteness). Red lines show the relationships summarised in part A of S3 Table. In the Europe plots, blue symbols and lines show the data (and relationships) for the United Kingdom National Parks.
(TIF)

S2 Fig The representativeness of our sample PAs. Histograms show the values of each of our predictor variables (top to bottom) for all terrestrial PAs in each region (left to right; excluding marine and IUCN Category I PAs), compared with the range represented in our sample of PAs (red vertical lines). For each predictor, the range of observed values is well covered by our sample, except for PA size, where we sampled no PAs <10 ha in area (black vertical lines); we therefore excluded these extremely small PAs from further analysis. Values for PA size, local population size, remoteness, and national income are all log_10_-transformed (after adding one to all values of mean visit rate, local population size, and remoteness).
(TIF)

S1 Table Annual visit data for PAs, 1998–2007. (DOCX)

S2 Table Univariate regressions between observed visit rates and potential predictor variables for each region. (DOCX)

S3 Table Results of GLMs of PA visit rates in each region. (DOCX)

S4 Table Empirically-derived estimates of the values of visits to terrestrial PAs or similar natural sites. (DOCX)

S1 Text Materials and methods. (DOCX)

## Abbreviations

PA: protected area
IUCN: International Union for Conservation of Nature
GLM: generalised linear model
WDPA: World Database on Protected Areas
