# Supplementary figures and images for: Walk on the Wild Side: Estimating the Global Magnitude of Visits to Protected Areas
Source: PLoS Biol. 2015 Feb 24;13(2):e1002074. doi: 10.1371/journal.pbio.1002074 (PMC4339837; doi:10.1371/journal.pbio.1002074)

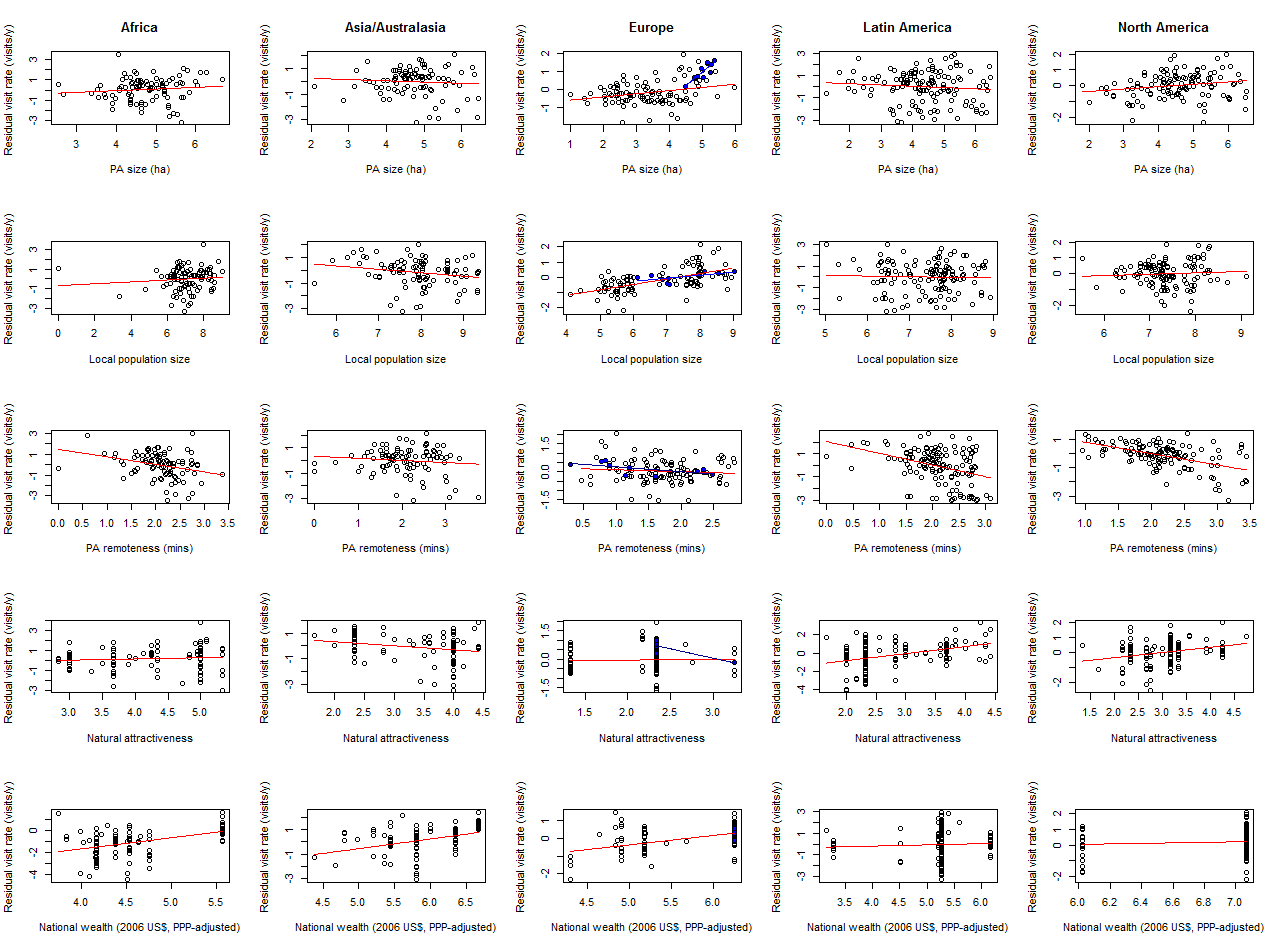

Supplement: S1 Fig — Plots show observed visit rates (adjusted for every other predictor variable) against each predictor variable (top to bottom) for each region (left to right). Values for mean visit rate, PA size, local population size, remoteness, and national income are all log10-transformed (after adding one to all values of mean visit rate and local population size and remoteness). Red lines show the relationships summarised in part A of S3 Table. In the Europe plots, blue symbols and lines show the data (and relationships) for the United Kingdom National Parks. (TIF) [file pbio.1002074.s001.tif]

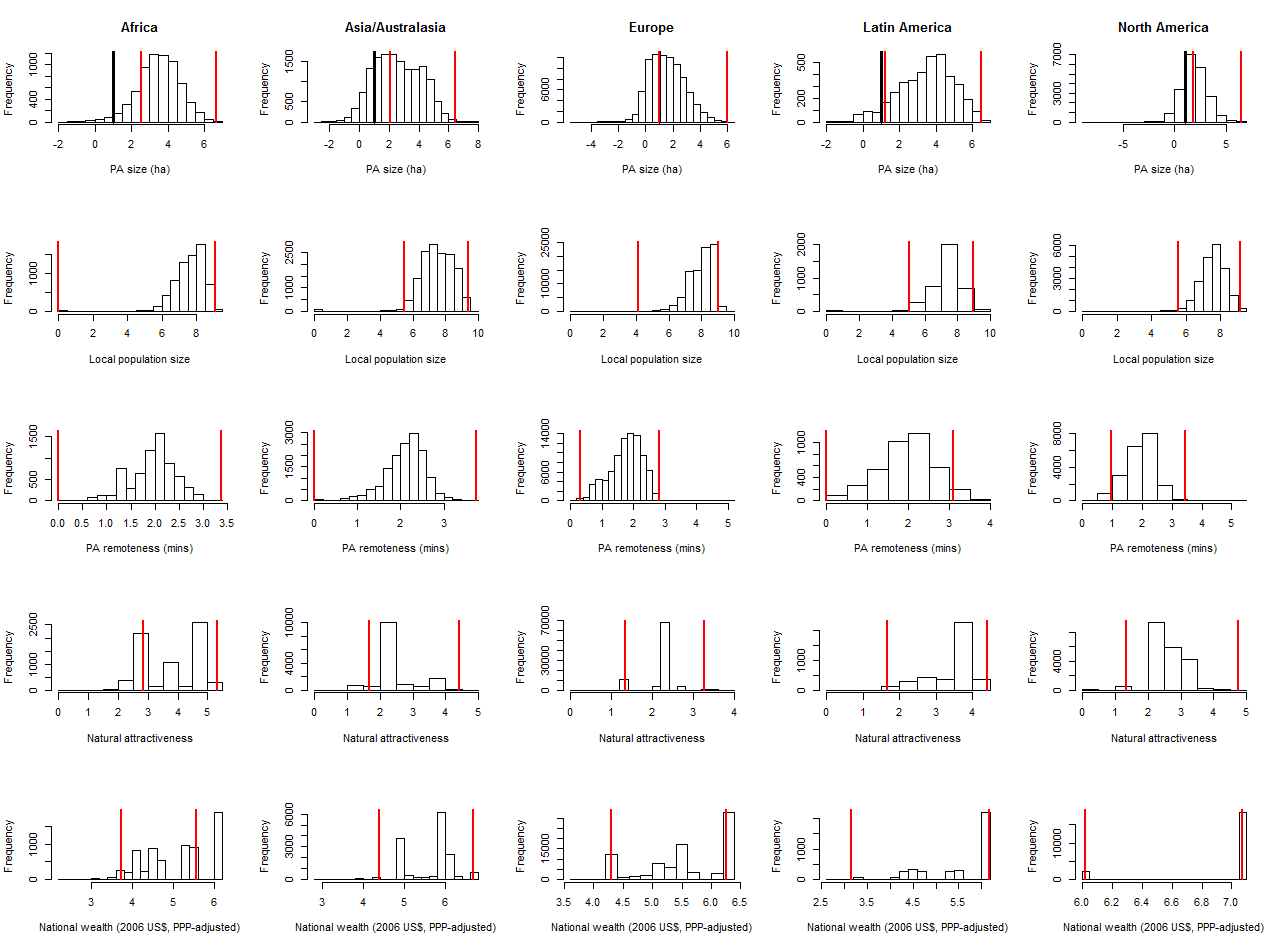

Supplement: S2 Fig — Histograms show the values of each of our predictor variables (top to bottom) for all terrestrial PAs in each region (left to right; excluding marine and IUCN Category I PAs), compared with the range represented in our sample of PAs (red vertical lines). For each predictor, the range of observed values is well covered by our sample, except for PA size, where we sampled no PAs <10 ha in area (black vertical lines); we therefore excluded these extremely small PAs from further analysis. Values for PA size, local population size, remoteness, and national income are all log10-transformed (after adding one to all values of mean visit rate, local population size, and remoteness). (TIF) [file pbio.1002074.s002.tif]
