# Supplementary material for: Walk on the Wild Side: Estimating the Global Magnitude of Visits to Protected Areas
Source: PLoS Biol. 2015 Feb 24;13(2):e1002074. doi: 10.1371/journal.pbio.1002074 (PMC4339837; doi:10.1371/journal.pbio.1002074)
Supplement: S1 Table — (DOCX) [file pbio.1002074.s003.docx]

|  |  |  | **Annual Visit Rate** | | | | | | | | | | |  |
| --- | --- | --- | --- | --- | --- | --- | --- | --- | --- | --- | --- | --- | --- | --- |
| **Region** | **Country** | **Protected area** | **1998** | **1999** | **2000** | **2001** | **2002** | **2003** | **2004** | **2005** | **2006** | **2007** | **Mean** | **Source** |
| Af | Botswana | Moremi Game Reserve | 49556 | 46707 | 30835 | 31073 | 39734 | 37378 | 38422 | 41513 | 37381 |  | 39178 | [32] |
| Af | Cameroon | Mbam and Djerem National Park |  |  |  |  |  |  |  |  |  | 0 | 0 | Craigie, I., pers. comm. |
| Af | Cameroon | Nki National Park |  |  |  |  |  |  |  |  |  | 0 | 0 | Craigie, I., pers. comm. |
| Af | Cameroon | Waza National Park |  |  |  |  |  |  |  |  |  | 6000 | 6000 | Craigie, I., pers. comm. |
| Af | DRC | Garamba National Park |  |  |  |  |  |  |  |  |  | 100 | 100 | Craigie, I., pers. comm. |
| Af | DRC | Kahuzi-Biega National Park |  |  |  |  |  |  |  |  |  | 1200 | 1200 | Craigie, I., pers. comm. |
| Af | Ethiopia | Bale Mountains National Park |  |  |  |  |  |  |  |  |  | 1170 | 1170 | Craigie, I., pers. comm. |
| Af | Ethiopia | Simien National Park | 150 |  |  |  |  |  |  |  |  |  | 150 | [33] |
| Af | Ghana | Ankasa Forest Reserve |  |  |  |  |  |  |  |  |  | 2000 | 2000 | Craigie, I., pers. comm. |
| Af | Ghana | Bomfobiri Wildlife Sanctuary |  |  |  |  |  |  |  |  |  | 95 | 95 | Craigie, I., pers. comm. |
| Af | Ghana | Bui National Park |  |  |  |  |  |  |  |  |  | 255 | 255 | Craigie, I., pers. comm. |
| Af | Ghana | Digya National Park |  |  |  |  |  |  |  |  |  | 10 | 10 | Craigie, I., pers. comm. |
| Af | Ghana | Gbele Resource Reserve |  |  |  |  |  |  |  |  |  | 57 | 57 | Craigie, I., pers. comm. |
| Af | Ghana | Kalakpa Resource Reserve |  |  |  |  |  |  |  |  |  | 100 | 100 | Craigie, I., pers. comm. |
| Af | Ghana | Kyabobo National Park |  |  |  |  |  |  |  |  |  | 78 | 78 | Craigie, I., pers. comm. |
| Af | Ghana | Mole National Park | 5604 | 5093 | 5425 | 5754 | 7295 | 7904 | 10427 | 12522 | 12734 | 13734 | 8649 | [34]; Umaru Farouk, D., pers comm. |
| Af | Ghana | Shai Hills Resource Reserve |  |  |  |  |  |  |  |  |  | 9626 | 9626 | Craigie, I., pers. comm. |
| Af | Kenya | Arabuko Sokoke Forest Reserve |  |  |  |  |  |  |  |  |  | 4000 | 4000 | Craigie, I., pers. comm. |
| Af | Madagascar | Ambohitantely | * | * | * | * | * | * | * | * | * |  | * | * |
| Af | Madagascar | Analamerana |  |  |  |  |  |  |  |  |  | 11 | 11 | Craigie, I., pers. comm. |
| Af | Madagascar | Andohahela |  | * | * | * | * | * | * | * | * |  | * | * |
| Af | Madagascar | Andranomena |  | * | * |  | * | * | * | * | * |  | * | * |
| Af | Madagascar | Andringitra | * | * | * | * | * | * | * | * | * |  | * | * |
| Af | Madagascar | Ankarafantsika |  |  | * | * | * | * | * | * | * |  | * | * |
| Af | Madagascar | Ankarana Special Reserve | * | * | * | * | * | * | * | * | * |  | * | * |
| Af | Madagascar | Bezaha Mahafaly | * | * | * | * | * | * | * | * | * |  | * | * |
| Af | Madagascar | Isalo | * | * | * | * | * | * | * | * | * |  | * | * |
| Af | Madagascar | Kalambatritra |  |  |  |  |  |  | * | * | * |  | * | * |
| Af | Madagascar | Kirindy Mitea |  |  |  | * | * | * | * | * | * |  | * | * |
| Af | Madagascar | Mananara-Nord | * |  | * |  |  | * | * | * | * |  | * | * |
| Af | Madagascar | Manombo |  | * |  | * | * | * | * | * | * |  | * | * |
| Af | Madagascar | Manongarivo |  |  | * | * | * | * | * | * | * |  | * | * |
| Af | Madagascar | Mantadia | * | * | * | * | * | * | * | * | * |  | * | * |
| Af | Madagascar | Marojejy | * | * | * | * | * | * | * | * | * |  | * | * |
| Af | Madagascar | Midongy du sud |  |  |  |  |  |  | * | * | * |  | * | * |
| Af | Madagascar | Montagne d'Ambre |  |  |  |  |  |  |  |  |  | 12723 | 12723 | Craigie, I., pers. comm. |
| Af | Madagascar | Ranomafana | * | * | * | * | * | * | * | * | * |  | * | * |
| Af | Madagascar | Tsimanampetsotsa |  |  |  | * | * | * | * | * | * |  | * | * |
| Af | Madagascar | Tsingy de Namoroka |  |  |  | * | * | * | * | * | * |  | * | * |
| Af | Madagascar | Zahamena |  |  | * |  | * | * | * | * | * |  | * | * |
| Af | Madagascar | Zombitse-Vohibasia |  |  |  |  |  | * | * | * | * |  | * | * |
| Af | Namibia | Bwabwata West Game Park |  |  |  |  |  |  |  |  |  | 17000 | 17000 | Craigie, I., pers. comm. |
| Af | Namibia | Mangetti Game Reserve |  |  |  |  |  |  |  |  |  | 0 | 0 | Craigie, I., pers. comm. |
| Af | Republic of the Congo | Nouabale-Ndoki National Park |  |  |  |  |  |  |  |  |  | 500 | 500 | Craigie, I., pers. comm. |
| Af | Rwanda | Akagera National Park | 687 | 1143 | 1709 | 3164 | 3677 | 7388 | 16471 |  |  |  | 4891 | [35] |
| Af | Rwanda | Nyungwe National Park |  |  |  |  |  |  |  |  |  | 4034 | 4034 | Craigie, I., pers. comm. |
| Af | Rwanda | Volcanoes National Park (PNV) | 0 | 417 | 1313 | 2155 | 5575 | 7224 | 8542 |  |  |  | 3604 | [35] |
| Af | Senegal | Djoudj National Park | 5000 |  |  |  |  |  |  |  |  |  | 5000 | [33] |
| Af | South Africa | Addo Elephant National Park |  |  |  |  |  |  |  |  |  | 172000 | 172000 | Craigie, I., pers. comm. |
| Af | South Africa | Agulhas National Park |  |  |  |  |  |  | 27022 | 23480 | 27460 |  | 25987 | Stevens, J. pers. comm. |
| Af | South Africa | Augrabies Falls National Park |  |  |  |  | 69535 | 57174 | 58209 | 61746 | 83269 |  | 65987 | Stevens, J., pers. comm. |
| Af | South Africa | Bontebok |  |  |  |  | 15638 | 16553 | 15074 | 8724 | 5599 |  | 12318 | Stevens, J., pers. comm. |
| Af | South Africa | Cape Peninsula National Park |  |  |  |  | 1289161 | 1438645 | 1494264 | 1479308 | 1611869 |  | 1462649 | Stevens, J., pers. comm. |
| Af | South Africa | De Hoop Nature Reserve |  |  |  |  |  |  |  |  |  | 15700 | 15700 | Craigie, I., pers. comm. |
| Af | South Africa | Gamkaberg Nature Reserve |  |  |  |  |  |  |  |  |  | 1000 | 1000 | Craigie, I., pers. comm. |
| Af | South Africa | Golden Gate |  |  |  |  | 24287 | 19935 | 21870 | 25101 | 32688 |  | 24776 | Stevens, J., pers. comm. |
| Af | South Africa | Kalahari Gemsbok |  |  |  |  |  | 21713 | 23709 | 21811 | 23955 |  | 22797 | Stevens, J., pers. comm. |
| Af | South Africa | Karoo National Park |  |  |  |  |  |  |  |  |  | 32000 | 32000 | Craigie, I., pers. comm. |
| Af | South Africa | Kruger National Park |  |  |  |  |  |  |  |  |  | 1000000 | 1000000 | Craigie, I., pers. comm. |
| Af | South Africa | Mapungubwe |  |  |  |  | 538 | 1197 |  | 20370 | 21775 |  | 10970 | Stevens, J., pers. comm. |
| Af | South Africa | Marakele |  |  |  |  | 11895 | 17276 | 18626 | 19143 | 20463 |  | 17481 | Stevens, J., pers. comm. |
| Af | South Africa | Mountain Zebra National Park |  |  |  |  |  |  |  |  |  | 16000 | 16000 | Craigie, I., pers. comm. |
| Af | South Africa | Namaqua |  |  |  |  |  |  | 3902 | 2804 | 10817 |  | 5841 | Stevens, J., pers. comm. |
| Af | South Africa | Richtersveld |  |  |  |  | 4489 | 3717 | 3331 | 725 | 3732 |  | 3199 | Stevens, J., pers. comm. |
| Af | South Africa | Vaalbos |  |  |  |  | 1818 | 2366 | 4461 | 1606 |  |  | 2563 | Stevens, J., pers. comm. |
| Af | South Africa | West Coast |  |  |  |  | 71259 | 46726 | 85798 | 140664 | 154185 |  | 99726 | Stevens, J., pers. comm. |
| Af | South Africa | Wilderness |  |  |  |  | 24952 | 36805 | 21471 | 16042 | 17620 |  | 23378 | Stevens, J., pers. comm. |
| Af | Tanzania | Arusha National Park | 19137 | 21731 | 21930 | 25372 | 22338 |  |  |  |  |  | 22102 | [36] |
| Af | Tanzania | Gombe National Park | 401 | 361 | 688 | 858 | 667 |  |  |  |  |  | 595 | [36] |
| Af | Tanzania | Katavi National Park | 368 | 325 | 325 | 1574 | 671 |  |  |  |  |  | 653 | [36] |
| Af | Tanzania | Kilimanjaro national park | 21940 | 21025 | 24775 | 25250 | 27852 |  |  |  |  |  | 24168 | [36] |
| Af | Tanzania | Lake Manyara National Park | 67805 | 67935 | 70273 | 71168 | 81285 |  |  |  |  |  | 71693 | [36] |
| Af | Tanzania | Mahale Mountain National Park | 217 | 196 | 271 | 916 | 586 |  |  |  |  |  | 437 | [36] |
| Af | Tanzania | Mikumi National Park | 10986 | 12347 | 11352 | 15766 | 15696 |  |  |  |  |  | 13229 | [36] |
| Af | Tanzania | Ruaha National Park | 5274 | 5646 | 6088 | 13094 | 6984 |  |  |  |  | 18000 | 9181 | [36]; Craigie, I., pers. comm. |
| Af | Tanzania | Rubondo National Park | 330 | 354 | 266 | 573 | 824 |  |  |  |  |  | 469 | [36] |
| Af | Tanzania | Selous Game Reserve | 5032 | 5844 | 5698 | 5284 |  |  |  |  |  |  | 5465 | [37] |
| Af | Tanzania | Serengeti National Park | 198934 | 113867 | 124553 | 149498 | 152544 |  |  |  |  |  | 147879 | [36] |
| Af | Tanzania | Tarangire National Park | 41147 | 48499 | 56871 | 68076 | 61077 |  |  |  |  |  | 55134 | [36] |
| Af | Tanzania | Udzungwa Mountain National Park | 483 | 751 | 1027 | 1455 | 1569 |  |  |  | 2433 |  | 1286 | [36] |
| Af | Uganda | Bwindi Impenetrable National Park | 3437 | 2100 | 3983 | 4517 | 5075 | 4900 |  |  |  | 8976 | 4713 | [38]; Craigie, I., pers. comm. |
| Af | Uganda | Kibale National Park |  |  |  |  |  |  |  |  |  | 6500 | 6500 | Craigie, I., pers. comm. |
| Af | Uganda | Kidepo Valley National Park | 1840 | 1501 | 2285 | 2470 | 1443 |  |  |  |  | 910 | 1742 | [38]; Craigie, I., pers. comm.. |
| Af | Uganda | Lake Mburo National Park | 8182 | 8552 | 8443 | 9616 | 10800 |  |  |  |  |  | 9119 | [38] |
| Af | Uganda | Mgahinga Gorilla Sanctuary | 2698 | 1741 | 2517 | 2205 | 1485 | 2506 |  |  |  |  | 2192 | [38] |
| Af | Uganda | Mt Elgon Nature Reserve | 1231 | 1308 | 1872 | 2024 | 3234 |  |  |  |  |  | 1934 | [38] |
| Af | Uganda | Murchison Falls National Park |  | 12687 | 23169 | 23578 | 27825 | 39262 | 46033 | 37133 |  | 29152 | 29855 | [39]; Craigie, I., pers. comm. |
| Af | Uganda | Queen Elizabeth National Park | 8349 | 8073 | 9231 | 14809 | 26632 |  |  |  |  | 47617 | 19119 | Balmford, A., pers. comm.; Craigie, I., pers. comm. |
| Af | Uganda | Rwenzori Mountains National Park |  |  |  | 117 | 268 |  |  |  |  |  | 193 | [38] |
| Af | Uganda | Rwenzori National Park |  |  |  |  |  |  |  |  |  | 1600 | 1600 | Craigie, I., pers. comm. |
| Af | Uganda | Semuliki National Park | 0 | 0 | 0 | 77 | 802 |  |  |  |  |  | 176 | [38] |
| Af | Zambia | Kasanka National Park |  |  |  |  |  |  |  |  |  | 1422 | 1422 | Craigie, I., pers. comm. |
| Af | Zambia | Lavushi Manda National Park |  |  |  |  |  |  |  |  |  | 0 | 0 | Craigie, I., pers. comm. |
| Af | Zimbabwe | Victoria Falls/Mosi-oa-Tunya |  |  | 300000 |  |  |  |  |  |  |  | 300000 | http://www.miombosafaricamp.com/special-features/victoria-falls (accessed 17/10/2014) |
| As/Au | Australia | Ben Lomond National Park | 32000 | 26200 | 32000 | 20000 | 23000 | 52000 | 54000 |  |  |  | 34171 | http://www.parks.tas.gov.au/index.aspx?base=866 (accessed 07/11/2014) |
| As/Au | Australia | Gondwana Rainforests of Australia |  |  | 510000 |  |  |  |  |  |  |  | 510000 | [40] |
| As/Au | Australia | Cradle Mountain- Lake St Clair | 204000 | 240000 | 225000 | 227583 | 264284 | 286620 | 283078 | 262930 | 269493 | 269804 | 253279 | http://www.parks.tas.gov.au/index.aspx?base=866 (accessed 07/11/2014) |
| As/Au | Australia | Douglas-Apsley National Park | 21000 | 21000 |  | 19000 |  |  |  |  |  |  | 20333 | http://www.parks.tas.gov.au/index.aspx?base=866 (accessed 07/11/2014) |
| As/Au | Australia | Franklin Gordon Wild Rivers | 101000 | 103000 | 106879 | 82300 | 95474 | 105532 | 93206 | 88031 | 103813 | 109000 | 98824 | http://www.parks.tas.gov.au/index.aspx?base=866 (accessed 07/11/2014) |
| As/Au | Australia | Freycinet National Park | 156000 | 187000 | 164000 | 169509 | 194081 | 197645 | 206436 | 203046 | 208045 | 207225 | 189299 | http://www.parks.tas.gov.au/index.aspx?base=866 (accessed 07/11/2014) |
| As/Au | Australia | Hartz Mountains National Park |  | 11000 | 11000 | 12000 | 12000 | 14000 | 11800 |  |  |  | 11967 | http://www.parks.tas.gov.au/index.aspx?base=866 (accessed 07/11/2014) |
| As/Au | Australia | Hastings Caves | 28000 | 27000 | 25000 | 28000 | 28000 | 30000 | 39000 | 40000 | 39000 | 37000 | 32100 | http://www.parks.tas.gov.au/index.aspx?base=866 (accessed 07/11/2014) |
| As/Au | Australia | Kakadu National Park |  |  | 200000 |  |  |  |  |  |  |  | 200000 | [40] |
| As/Au | Australia | Litchfield National Park |  | 273000 |  |  |  |  |  |  |  |  | 273000 | [33] |
| As/Au | Australia | Mole Creek Karst National Park | 50395 | 50660 | 45370 | 44082 | 48630 | 45423 | 43568 | 46809 | 46018 | 46388 | 46734 | http://www.parks.tas.gov.au/index.aspx?base=866 (accessed 07/11/2014) |
| As/Au | Australia | Moreton Island National Park |  |  | 75000 |  |  |  |  |  |  |  | 75000 | http://www.fido.org.au/moonbi/backgrounders/11%20Carry%20Capacity.pdf (accessed 07/11/2014) |
| As/Au | Australia | Mt Field National Park | 141000 | 130700 | 138700 | 105102 | 116398 | 130855 | 128959 | 116547 | 109347 | 115490 | 123310 | http://www.parks.tas.gov.au/index.aspx?base=866 (accessed 07/11/2014) |
| As/Au | Australia | Purnululu National Park |  |  | 17000 |  |  |  |  |  |  |  | 17000 | [40] |
| As/Au | Australia | South Bruny National Park |  |  | 24000 | 25000 | 28000 | 36000 | 34000 |  |  |  | 29400 | http://www.parks.tas.gov.au/index.aspx?base=866 (accessed 07/11/2014) |
| As/Au | Australia | Southwest National Park |  |  |  | 35000 | 60000 | 65000 | 61000 | 27000 | 30000 |  | 46333 | http://www.parks.tas.gov.au/index.aspx?base=866 (accessed 07/11/2014) |
| As/Au | Australia | Uluru-Kata Tjuta |  |  | 380000 |  |  |  |  |  |  |  | 380000 | [40] |
| As/Au | Australia | Walls of Jerusalem National Park | 3000 | 3000 | 3000 | 3000 | 3000 | 3000 | 3000 | 3000 | 3000 | 3000 | 3000 | http://www.parks.tas.gov.au/index.aspx?base=866 (accessed 07/11/2014) |
| As/Au | China | Dafeng Milu Nature Reserve | 119800 | 120984 | 130045 | 130953 | 157042 | 189250 | 251390 | 325703 |  |  | 178146 | del Mar Otero Villanueva, M., pers. comm. |
| As/Au | China | Huanglong | 260000 |  |  | 823254 |  |  |  |  |  |  | 541627 | [41]) |
| As/Au | China | Jiuzhaigou | 200000 |  |  |  |  |  |  |  |  |  | 200000 | [33] |
| As/Au | China | Mount Emei Scenic Area | 300000 |  |  |  |  |  |  |  |  |  | 300000 | [33] |
| As/Au | China | Mount Huangshan |  |  |  | 1340000 |  |  |  |  |  |  | 1340000 | [42] |
| As/Au | China | Sichuan Giant Panda Sanctuaries |  |  | 400000 |  |  |  |  |  |  |  | 400000 | [43] |
| As/Au | China | Three Parallel Rivers of Yunnan Protected Areas |  |  |  | 188560 |  |  |  |  |  |  | 188560 | [44] |
| As/Au | China | Wolong Nature Reserve | 52400 | 66700 | 108100 | 90000 | 82000 | 66000 | 163400 | 206100 | 235500 | 115100 | 118530 | [45] |
| As/Au | China | Wulingyuan Scenic Area | 350000 |  |  |  |  |  |  |  |  |  | 350000 | [33] |
| As/Au | China | Wuyishan National Park |  |  |  |  |  | 50672 | 45195 | 34725 |  |  | 43531 | Haohan, W., pers. comm. |
| As/Au | China | Wuyishan Scenic Area |  |  |  |  |  | 1055000 | 1200000 | 1543000 |  |  | 1266000 | Haohan, W., pers. comm. |
| As/Au | India | Bandhavgarh |  |  |  |  |  |  | 25306 |  |  |  | 25306 | [46] |
| As/Au | India | Bandipur |  |  |  |  |  |  | 51986 |  |  |  | 51986 | [47] |
| As/Au | India | Bhadra |  |  |  |  |  |  | 1316 |  |  |  | 1316 | [46] |
| As/Au | India | Corbett |  |  |  |  |  |  | 95220 |  |  |  | 95220 | [47] |
| As/Au | India | Jaldapara Wildlife Sanctuary |  |  |  |  |  |  | 2778 |  |  |  | 2778 | [48] |
| As/Au | India | Kalakad |  |  |  |  |  |  | 70807 |  |  |  | 70807 | [47] |
| As/Au | India | Kanha |  |  |  |  |  |  | 70464 |  |  |  | 70464 | [47] |
| As/Au | India | Kaziranga National Park | 19525 | 19248 | 39319 | 52336 | 46306 | 61866 | 61637 | 72873 | 77073 |  | 50020 | Balmford, A., pers. comm. |
| As/Au | India | Ken Gharial Sanctuary |  |  |  |  |  |  | 32069 |  |  |  | 32069 | [46] |
| As/Au | India | Melghat |  |  |  |  |  |  | 26997 |  |  |  | 26997 | [47] |
| As/Au | India | Mudumalai |  |  |  |  |  |  | 94484 |  |  |  | 94484 | [46] |
| As/Au | India | Nagarahole |  |  |  |  |  |  | 36844 |  |  |  | 36844 | [46] |
| As/Au | India | Panna |  |  |  |  |  |  | 36404 |  |  |  | 36404 | [47] |
| As/Au | India | Pench |  |  |  |  |  |  | 32146 |  |  |  | 32146 | [46] |
| As/Au | India | Periyar |  |  |  |  |  |  | 415373 |  |  |  | 415373 | [47] |
| As/Au | India | Ranthambore |  |  |  |  | 54817 | 48653 |  |  |  |  | 51735 | Behl, A., pers. comm. |
| As/Au | India | Sariska |  |  |  |  |  |  | 49451 |  |  |  | 49451 | [47] |
| As/Au | India | Satpura |  |  |  |  |  |  | 162785 |  |  |  | 162785 | [47] |
| As/Au | India | The Sundarbans |  |  |  |  |  |  | 60000 |  |  |  | 60000 | [33]; [47] |
| As/Au | India | Valley of Flowers National Park | 420 | 750 | 1220 | 2900 | 2970 | 4450 | 4850 |  |  |  | 2509 | [49] |
| As/Au | Indonesia | Alas Purwo |  |  |  | 11580 | 2048 | 8624 | 17614 | 11643 |  |  | 10302 | http://www.dephut.go.id/INFORMASI/STATISTIK/2005/II_1_2.pdf (accessed 14/11/2014) |
| As/Au | Indonesia | Betung Kerihun |  |  |  | 130 |  | 18 | 55 | 50 |  |  | 63 | http://www.dephut.go.id/INFORMASI/STATISTIK/2005/II_1_2.pdf (accessed 14/11/2014) |
| As/Au | Indonesia | Bogani Nani Wartabone |  |  |  | 500 |  |  | 32 | 540 |  |  | 357 | http://www.dephut.go.id/INFORMASI/STATISTIK/2005/II_1_2.pdf (accessed 14/11/2014) |
| As/Au | Indonesia | Bromo Tengger Semeru | 130620 | 141135 |  | 76590 | 155846 | 101727 | 13235 | 86669 |  |  | 100832 | [50]; http://www.dephut.go.id/INFORMASI/STATISTIK/2005/II_1_2.pdf (accessed 14/11/2014) |
| As/Au | Indonesia | Bukit Duabelas |  |  |  |  | 10 |  |  |  |  |  | 10 | http://www.dephut.go.id/INFORMASI/STATISTIK/2005/II_1_2.pdf (accessed 14/11/2014) |
| As/Au | Indonesia | Bukit Tigapuluh |  |  |  |  |  | 5761 | 221 |  |  |  | 2991 | http://www.dephut.go.id/INFORMASI/STATISTIK/2005/II_1_2.pdf (accessed 14/11/2014) |
| As/Au | Indonesia | Danau Sentarum |  |  |  |  |  |  | 13 |  |  |  | 13 | http://www.dephut.go.id/INFORMASI/STATISTIK/2005/II_1_2.pdf (accessed 14/11/2014) |
| As/Au | Indonesia | Gunung Ciremai |  |  |  |  |  |  |  | 28540 |  |  | 28540 | http://www.dephut.go.id/INFORMASI/STATISTIK/2005/II_1_2.pdf (accessed 14/11/2014) |
| As/Au | Indonesia | Gunung Gede Pangrango |  |  |  | 129285 | 52174 | 6476 | 57329 |  |  |  | 61316 | http://www.dephut.go.id/INFORMASI/STATISTIK/2005/II_1_2.pdf (accessed 14/11/2014) |
| As/Au | Indonesia | Gunung Halimun |  |  |  | 4888 | 9589 | 3031 | 4308 | 3149 |  |  | 4993 | http://www.dephut.go.id/INFORMASI/STATISTIK/2005/II_1_2.pdf (accessed 14/11/2014) |
| As/Au | Indonesia | Gunung Leuser |  |  |  | 9352 |  |  |  |  |  |  | 9352 | http://www.dephut.go.id/INFORMASI/STATISTIK/2005/II_1_2.pdf (accessed 14/11/2014) |
| As/Au | Indonesia | Gunung Palung |  |  |  | 1223 | 167 | 81 | 4 |  |  |  | 369 | http://www.dephut.go.id/INFORMASI/STATISTIK/2005/II_1_2.pdf (accessed 14/11/2014) |
| As/Au | Indonesia | Gunung Rinjani |  |  |  | 56681 | 63799 | 27945 |  | 86971 |  |  | 58849 | http://www.dephut.go.id/INFORMASI/STATISTIK/2005/II_1_2.pdf (accessed 14/11/2014) |
| As/Au | Indonesia | Kelimutu |  |  |  | 6466 | 1152 | 6674 | 1419 | 5749 |  |  | 4292 | http://www.dephut.go.id/INFORMASI/STATISTIK/2005/II_1_2.pdf (accessed 14/11/2014) |
| As/Au | Indonesia | Kerinci Seblat |  |  |  | 1704 |  | 1127 |  |  |  |  | 1416 | http://www.dephut.go.id/INFORMASI/STATISTIK/2005/II_1_2.pdf (accessed 14/11/2014) |
| As/Au | Indonesia | Lore Lindu |  |  |  |  |  |  | 39 |  |  |  | 39 | http://www.dephut.go.id/INFORMASI/STATISTIK/2005/II_1_2.pdf (accessed 14/11/2014) |
| As/Au | Indonesia | Lorentz |  |  |  | 2 | 9 |  | 32 |  |  |  | 14 | http://www.dephut.go.id/INFORMASI/STATISTIK/2005/II_1_2.pdf (accessed 14/11/2014) |
| As/Au | Indonesia | Siberut |  |  |  | 49 |  |  |  |  |  |  | 49 | http://www.dephut.go.id/INFORMASI/STATISTIK/2005/II_1_2.pdf (accessed 14/11/2014) |
| As/Au | Korea | Hallasan National Park |  |  |  | 450000 | 425000 | 557000 | 668000 | 734000 | 745000 | 804000 | 626143 | http://www.npa.or.kr/www/eng/npd/snp/num01.htm (accessed 18/11/2014) |
| As/Au | Korea | Seoraksan National Park |  |  |  | 2976000 | 2883000 | 2970000 | 3300000 | 3125000 | 2678000 | 3490000 | 3060286 | http://www.npa.or.kr/www/eng/npd/snp/num01.htm (accessed 18/11/2014) |
| As/Au | Korea | Sobaeksan National Park |  |  |  | 273000 | 294000 | 337000 | 387000 | 392000 | 356000 | 460000 | 357000 | http://www.npa.or.kr/www/eng/npd/snp/num01.htm (accessed 18/11/2014) |
| As/Au | Malaysia | Batang Ai National Park |  |  |  |  |  |  |  |  | 8750 |  | 8750 | [51] |
| As/Au | Malaysia | Endau Rompin National Park |  |  |  |  |  |  |  |  | 9439 |  | 9439 | [51] |
| As/Au | Malaysia | Gunung Gading National Park |  |  |  |  |  |  |  |  | 7230 |  | 7230 | [51] |
| As/Au | Malaysia | Kinabalu National Park | 370000 |  |  |  |  |  |  |  |  |  | 370000 | [52] |
| As/Au | Malaysia | Kubah National Park |  |  |  |  |  |  |  |  | 5271 |  | 5271 | [51] |
| As/Au | Malaysia | Lambur Hills National Park |  |  |  |  |  |  |  |  | 16007 |  | 16007 | [51] |
| As/Au | Malaysia | Loagan Bunut National Park |  |  |  |  |  |  |  |  | 2974 |  | 2974 | [51] |
| As/Au | Malaysia | Niah National Park |  |  |  |  |  |  |  |  | 9000 |  | 9000 | [51] |
| As/Au | Malaysia | Taman Negara national Park |  |  |  |  |  |  |  |  | 65000 |  | 65000 | [51] |
| As/Au | Malaysia | Tanjung Datu National Park |  |  |  |  |  |  |  |  | 197 |  | 197 | [51] |
| As/Au | Malaysia | Tawau Hills Park |  |  |  |  |  |  |  |  | 21250 |  | 21250 | [51] |
| As/Au | Nepal | Shey Phoksundo National Park |  |  |  |  |  |  |  |  |  | 500 | 500 | Craigie, I., pers. comm. |
| As/Au | New Zealand | Abel Tasman National Park |  |  |  |  |  |  | 190000 |  |  |  | 190000 | [53] |
| As/Au | Oman | Arabian Oryx Sanctuary | 150 |  |  |  |  |  |  |  |  |  | 150 | [33] |
| As/Au | Philippines | Mount Kitanglad Natural Park |  |  |  |  |  |  |  |  |  | 800 | 800 | Craigie, I., pers. comm. |
| As/Au | Philippines | Mount Pulag National Park |  |  |  |  |  |  |  |  |  | 3365 | 3365 | Craigie, I., pers. comm. |
| As/Au | Philippines | Puerta-Princesa Subterranean River National Park | 30776 | 31201 | 32639 | 22613 | 18233 | 26229 | 34596 | 34142 | 46268 | 63271 | 33997 | http://www.puerto-undergroundriver.com/tourism/visitor/ (accessed 17/11/2014); Mallari, N.A., pers. comm. |
| As/Au | Sri Lanka | Horton Plains | * | * | * | * | * | * | * | * |  |  | * | * |
| As/Au | Sri Lanka | Uda Walawe | * | * | * | * | * | * | * | * |  |  | * | * |
| As/Au | Sri Lanka | Wasgomuwa | * | * | * | * | * | * | * | * |  |  | * | * |
| As/Au | Sri Lanka | Yala | * | * | * | * | * | * | * | * |  |  | * | * |
| As/Au | Thailand | Dong Phayayan Khao-Yai Forest Complex |  |  |  |  | 784370 |  |  |  |  |  | 784370 | [54] |
| As/Au | UAE | Dubai Desert Conservation Reserve |  |  |  |  |  |  | 167245 | 204529 | 211525 |  | 194433 | Simkins, G., pers. comm. |
| As/Au | Vietnam | Cuc Phuong National Park |  |  | 49350 |  |  | 60463 |  |  |  |  | 54907 | Nguyen, Q., pers. comm. |
| As/Au | Vietnam | Phong Nha-Ke Bang National Park |  | 81400 |  |  |  |  |  |  |  |  | 81400 | [55] |
| As/Au | Vietnam | Pu Luong Nature Reserve |  |  |  |  |  |  |  |  |  | 1000 | 1000 | Craigie, I., pers. comm. |
| Eu | Belarus | Belovezhskaya Pushcha | 80000 |  |  |  |  |  |  |  |  |  | 80000 | [33] |
| Eu | Bulgaria | Central Balkan National Park |  |  |  |  |  |  |  |  |  | 50000 | 50000 | Craigie, I., pers. comm. |
| Eu | Bulgaria | Pirin National Park | 400000 |  |  |  |  |  |  |  |  |  | 400000 | [33] |
| Eu | Bulgaria | Vitosha Nature Park |  |  |  |  |  |  |  |  |  | 3500000 | 3500000 | Craigie, I., pers. comm. |
| Eu | Estonia | Endla National Park | 2277 |  |  |  |  |  |  |  |  |  | 2277 | http://enrin.grida.no/biodiv/biodiv/national/estonia/press3.htm (accessed 17/11/2014) |
| Eu | Estonia | Haanja | 300 |  |  |  |  |  |  |  |  |  | 300 | http://enrin.grida.no/biodiv/biodiv/national/estonia/press3.htm (accessed 17/11/2014) |
| Eu | Estonia | Karula National Park | 2500 |  |  |  |  |  |  |  |  |  | 2500 | http://enrin.grida.no/biodiv/biodiv/national/estonia/press3.htm (accessed 17/11/2014) |
| Eu | Estonia | Soomaa National Park | 2900 |  |  |  |  |  |  |  |  |  | 2900 | http://enrin.grida.no/biodiv/biodiv/national/estonia/press3.htm (accessed 17/11/2014) |
| Eu | Finland | Helvetinjärven kansallispuisto |  |  |  |  | 32000 | 32000 | 32000 | 32000 | 32000 | 33000 | 32167 | http://julkaisut.metsa.fi/julkaisut/?Text=Natural%20Heritage%20Services%20Annual%20Report&col=3&dir=DESC (accessed 17/11/2014) |
| Eu | Finland | Hiidenportin kansallispuisto |  |  |  |  | 8000 | 7500 | 7700 | 10000 | 10000 | 6500 | 8283 | http://julkaisut.metsa.fi/julkaisut/?Text=Natural%20Heritage%20Services%20Annual%20Report&col=3&dir=DESC (accessed 17/11/2014) |
| Eu | Finland | Isojärven kansallispuisto |  |  |  |  | 8000 | 8000 | 9000 | 8000 | 7000 | 8000 | 8000 | http://julkaisut.metsa.fi/julkaisut/?Text=Natural%20Heritage%20Services%20Annual%20Report&col=3&dir=DESC (accessed 17/11/2014) |
| Eu | Finland | Kauhanevan-Pohjankankaan kansallispuisto |  |  |  |  | 6000 | 6000 | 6000 | 6000 | 6000 | 6000 | 6000 | http://julkaisut.metsa.fi/julkaisut/?Text=Natural%20Heritage%20Services%20Annual%20Report&col=3&dir=DESC (accessed 17/11/2014) |
| Eu | Finland | Kurjenrahkan kansallispuisto |  |  |  | 20000 |  | 20000 | 20000 | 25000 | 25000 | 32500 | 23750 | http://julkaisut.metsa.fi/julkaisut/?Text=Natural%20Heritage%20Services%20Annual%20Report&col=3&dir=DESC (accessed 17/11/2014) |
| Eu | Finland | Lauhanvuoren kansallispuisto |  |  |  |  | 30000 | 25000 | 27000 | 27000 | 27000 | 27500 | 27250 | http://julkaisut.metsa.fi/julkaisut/?Text=Natural%20Heritage%20Services%20Annual%20Report&col=3&dir=DESC (accessed 17/11/2014) |
| Eu | Finland | Leivonmäen kansallispuisto |  |  |  |  |  | 4500 | 7000 | 10000 | 11000 | 12000 | 8900 | http://julkaisut.metsa.fi/julkaisut/?Text=Natural%20Heritage%20Services%20Annual%20Report&col=3&dir=DESC (accessed 17/11/2014) |
| Eu | Finland | Lemmenjoen kansallispuisto |  |  |  |  | 10000 | 10000 | 10000 | 10000 | 10000 | 10000 | 10000 | http://julkaisut.metsa.fi/julkaisut/?Text=Natural%20Heritage%20Services%20Annual%20Report&col=3&dir=DESC (accessed 17/11/2014) |
| Eu | Finland | Liesjärven kansallispuisto |  |  |  | 25000 |  | 15000 | 16000 | 25000 | 25000 | 22000 | 21333 | http://julkaisut.metsa.fi/julkaisut/?Text=Natural%20Heritage%20Services%20Annual%20Report&col=3&dir=DESC (accessed 17/11/2014) |
| Eu | Finland | Nuuksion kansallispuisto |  |  |  | 100000 |  | 100000 | 100000 | 110000 | 142000 | 170000 | 120333 | http://julkaisut.metsa.fi/julkaisut/?Text=Natural%20Heritage%20Services%20Annual%20Report&col=3&dir=DESC (accessed 17/11/2014) |
| Eu | Finland | Oulangan kansallispuisto |  |  |  |  | 162000 | 165000 | 173000 | 173500 | 183500 | 185500 | 173750 | http://julkaisut.metsa.fi/julkaisut/?Text=Natural%20Heritage%20Services%20Annual%20Report&col=3&dir=DESC (accessed 17/11/2014) |
| Eu | Finland | Päijänteen kansallispuisto |  |  |  |  | 8000 | 8000 | 10000 | 12000 | 12000 | 12000 | 10333 | http://julkaisut.metsa.fi/julkaisut/?Text=Natural%20Heritage%20Services%20Annual%20Report&col=3&dir=DESC (accessed 17/11/2014) |
| Eu | Finland | Pallas-Yllästunturin kansallispuisto |  |  |  |  |  |  |  | 300000 | 310000 | 312000 | 307333 | http://julkaisut.metsa.fi/julkaisut/?Text=Natural%20Heritage%20Services%20Annual%20Report&col=3&dir=DESC (accessed 17/11/2014) |
| Eu | Finland | Patvinsuon kansallispuisto |  |  |  | 15000 |  | 15000 | 20000 | 14000 | 15000 | 14000 | 15500 | http://julkaisut.metsa.fi/julkaisut/?Text=Natural%20Heritage%20Services%20Annual%20Report&col=3&dir=DESC (accessed 17/11/2014) |
| Eu | Finland | Petkeljärven kansallispuisto |  |  |  | 15000 |  | 17000 | 17000 | 17500 | 18500 | 23000 | 18000 | http://julkaisut.metsa.fi/julkaisut/?Text=Natural%20Heritage%20Services%20Annual%20Report&col=3&dir=DESC (accessed 17/11/2014) |
| Eu | Finland | Pyhä-Häkin kansallispuisto |  |  |  |  | 11000 | 11000 | 11000 | 9000 | 15500 | 14500 | 12000 | http://julkaisut.metsa.fi/julkaisut/?Text=Natural%20Heritage%20Services%20Annual%20Report&col=3&dir=DESC (accessed 17/11/2014) |
| Eu | Finland | Pyhä-Luoston kansallispuisto |  |  |  |  |  |  |  | 95000 | 103500 | 109500 | 102667 | http://julkaisut.metsa.fi/julkaisut/?Text=Natural%20Heritage%20Services%20Annual%20Report&col=3&dir=DESC (accessed 17/11/2014) |
| Eu | Finland | Repoveden kansallispuisto |  |  |  |  |  | 65000 | 65000 | 65000 | 69000 | 70000 | 66800 | http://julkaisut.metsa.fi/julkaisut/?Text=Natural%20Heritage%20Services%20Annual%20Report&col=3&dir=DESC (accessed 17/11/2014) |
| Eu | Finland | Riisitunturin kansallispuisto |  |  |  |  | 6000 | 7000 | 7000 | 7000 | 7000 | 8000 | 7000 | http://julkaisut.metsa.fi/julkaisut/?Text=Natural%20Heritage%20Services%20Annual%20Report&col=3&dir=DESC (accessed 17/11/2014) |
| Eu | Finland | Rokuan kansallispuisto |  |  |  |  | 24000 | 24000 | 20000 | 20000 | 18000 | 23500 | 21583 | http://julkaisut.metsa.fi/julkaisut/?Text=Natural%20Heritage%20Services%20Annual%20Report&col=3&dir=DESC (accessed 17/11/2014) |
| Eu | Finland | Salamajärven kansallispuisto |  |  |  |  | 7000 | 7000 | 9000 | 10000 | 12000 | 11000 | 9333 | http://julkaisut.metsa.fi/julkaisut/?Text=Natural%20Heritage%20Services%20Annual%20Report&col=3&dir=DESC (accessed 17/11/2014) |
| Eu | Finland | Seitsemisen kansallispuisto |  |  |  |  | 37000 | 40000 | 40000 | 40000 | 42000 | 44000 | 40500 | http://julkaisut.metsa.fi/julkaisut/?Text=Natural%20Heritage%20Services%20Annual%20Report&col=3&dir=DESC (accessed 17/11/2014) |
| Eu | Finland | Syätteen kansallispuisto |  |  |  |  | 25000 | 24000 | 34000 | 33500 | 33000 | 36000 | 30917 | http://julkaisut.metsa.fi/julkaisut/?Text=Natural%20Heritage%20Services%20Annual%20Report&col=3&dir=DESC (accessed 17/11/2014) |
| Eu | Finland | Tiilikkajärven kansallispuisto |  |  |  |  | 6000 | 6000 | 7000 | 6500 | 7000 | 7000 | 6583 | http://julkaisut.metsa.fi/julkaisut/?Text=Natural%20Heritage%20Services%20Annual%20Report&col=3&dir=DESC (accessed 17/11/2014) |
| Eu | Finland | Torronsuon kansallispuisto |  |  |  | 15000 |  | 20000 | 20000 | 20000 | 20000 | 27000 | 20333 | http://julkaisut.metsa.fi/julkaisut/?Text=Natural%20Heritage%20Services%20Annual%20Report&col=3&dir=DESC (accessed 17/11/2014) |
| Eu | Finland | Urho Kekkosen kansallispuisto |  |  |  |  | 150000 | 160000 | 160000 | 165000 | 170000 | 180000 | 164167 | http://julkaisut.metsa.fi/julkaisut/?Text=Natural%20Heritage%20Services%20Annual%20Report&col=3&dir=DESC (accessed 17/11/2014) |
| Eu | Finland | Valkmusan kansallispuisto |  |  |  | 6000 |  | 5000 | 5000 | 6000 | 6500 | 6200 | 5783 | http://julkaisut.metsa.fi/julkaisut/?Text=Natural%20Heritage%20Services%20Annual%20Report&col=3&dir=DESC (accessed 17/11/2014) |
| Eu | Hungary | Caves of Aggtelek Karst and Slovak Karst | 200000 |  |  |  |  |  |  |  |  |  | 200000 | [33] |
| Eu | Poland | Bialowieza National Park |  |  |  |  |  |  |  |  |  | 89476 | 89476 | Craigie, I., pers. comm. |
| Eu | Romania | Macin Mountains (Muntii Mæcinului Natural Park) |  |  |  |  |  |  |  |  |  | 4500 | 4500 | Craigie, I., pers. comm. |
| Eu | Slovakia | Biele Karpathy |  |  |  |  |  |  |  |  |  | 7605 | 7605 | Craigie, I., pers. comm. |
| Eu | Slovakia | Cerova vrchovina landscape |  |  |  |  |  |  |  |  |  | 21000 | 21000 | Craigie, I., pers. comm. |
| Eu | Slovakia | Horna Orava Protected Landscape |  |  |  |  |  |  |  |  |  | 35000 | 35000 | Craigie, I., pers. comm. |
| Eu | Slovakia | Kysuce Protected Landscape |  |  |  |  |  |  |  |  |  | 200000 | 200000 | Craigie, I., pers. comm. |
| Eu | Slovakia | Mala Fatra National Park |  |  |  |  |  |  |  |  |  | 1000000 | 1000000 | Craigie, I., pers. comm. |
| Eu | Slovakia | Muranska planina National Park |  |  |  |  |  |  |  |  |  | 20000 | 20000 | Craigie, I., pers. comm. |
| Eu | Slovakia | Pieninsky National Park |  |  |  |  |  |  |  |  |  | 300000 | 300000 | Craigie, I., pers. comm. |
| Eu | Slovakia | Polana Protected Landscape |  |  |  |  |  |  |  |  |  | 18000 | 18000 | Craigie, I., pers. comm. |
| Eu | Slovakia | Poloniny National Park |  |  |  |  |  |  |  |  |  | 20000 | 20000 | Craigie, I., pers. comm. |
| Eu | Slovakia | Slovensky raj National Park |  |  |  |  |  |  |  |  |  | 400000 | 400000 | Craigie, I., pers. comm. |
| Eu | Slovakia | Stiavnicke vrchy Protected Landscape |  |  |  |  |  |  |  |  |  | 50000 | 50000 | Craigie, I., pers. comm. |
| Eu | Slovakia | Strazovske vrchy Protected Landscape |  |  |  |  |  |  |  |  |  | 10000 | 10000 | Craigie, I., pers. comm. |
| Eu | Slovakia | Tatransky National Park |  |  |  |  |  |  |  |  |  | 2500000 | 2500000 | Craigie, I., pers. comm. |
| Eu | Slovenia | Skocjan Caves |  |  |  | 57000 | 66000 |  |  |  |  |  | 61500 | [56] |
| Eu | Sweden | Lapponian Area | 75000 |  |  |  |  |  |  |  |  |  | 75000 | [33] |
| Eu | UK | Arne | 33487 | 62810 | 26818 | 20700 | 44500 |  | 41725 | 64755 | 58778 |  | 44197 | Burton, G., pers. comm. |
| Eu | UK | Aylesbeare | 2500 | 10800 | 8500 | 4200 | 10970 |  |  | 16000 | 16000 |  | 9853 | Burton, G., pers. comm. |
| Eu | UK | Berney Marshes | 150 |  |  |  | 32 |  |  | 5000 | 5000 |  | 2546 | Burton, G., pers. comm. |
| Eu | UK | Blacktoft Sands | 22670 | 27304 | 19369 | 16269 | 19589 |  | 19682 | 20568 | 21036 |  | 20811 | Burton, G., pers. comm. |
| Eu | UK | Blean Woods | 8603 | 8915 | 6700 | 5064 | 6993 |  |  | 12295 | 12349 |  | 8703 | Burton, G., pers. comm. |
| Eu | UK | Campfield Marsh |  |  |  |  |  |  |  | 8500 | 10000 |  | 9250 | Burton, G., pers. comm. |
| Eu | UK | Coombes Valley | 9360 | 8636 | 4162 | 938 | 2538 |  | 12000 | 12000 | 12000 |  | 7704 | Burton, G., pers. comm. |
| Eu | UK | Dartmoor |  |  |  |  |  |  |  | 4300000 |  |  | 4300000 | [57] |
| Eu | UK | Dee Estuary | 7675 | 6181 | 6849 | 3657 | 8940 |  | 8175 | 8889 | 7643 |  | 7251 | Burton, G., pers. comm. |
| Eu | UK | Dungeness | 26176 | 25518 | 19053 | 19053 | 25255 |  | 28671 | 28505 | 29635 |  | 25233 | Burton, G., pers. comm. |
| Eu | UK | Elmley Marshes | 10604 | 9343 | 13621 | 3791 | 11613 |  | 11600 | 11000 | 12500 |  | 10509 | Burton, G., pers. comm. |
| Eu | UK | Exe Estuary |  |  |  |  |  |  | 10000 |  |  |  | 10000 | Burton, G., pers. comm. |
| Eu | UK | Exmoor |  |  |  |  |  |  |  | 800000 |  |  | 800000 | [57] |
| Eu | UK | Fairburn Ings | 56202 | 42601 | 59900 | 22415 | 24523 |  | 29665 | 23957 | 40666 |  | 37491 | Burton, G., pers. comm. |
| Eu | UK | Fowlmere | 20800 | 29890 | 23400 | 9600 | 30600 |  | 26900 | 21000 | 20700 |  | 22861 | Burton, G., pers. comm. |
| Eu | UK | Frampton | 1782 | 2460 | 1310 | 5718 | 7424 |  |  | 13573 | 9087 |  | 5908 | Burton, G., pers. comm. |
| Eu | UK | Garston Wood |  |  |  |  |  |  |  | 4000 | 4000 |  | 4000 | Burton, G., pers. comm. |
| Eu | UK | Geltsdale |  |  |  |  |  |  |  | 5000 | 5000 |  | 5000 | Burton, G., pers. comm. |
| Eu | UK | Giant's Causeway | 350000 |  |  |  |  |  |  |  |  |  | 350000 | [33] |
| Eu | UK | Ham Wall | 625 | 1880 | 2290 | 755 | 6050 |  |  | 19800 | 35600 |  | 9571 | Burton, G., pers. comm. |
| Eu | UK | Havergate Island | 384 | 437 |  | 120 | 144 |  |  |  |  |  | 271 | Burton, G., pers. comm. |
| Eu | UK | Haweswater | 5310 | 4574 | 6492 |  | 4358 |  | 3529 | 2532 | 3836 |  | 4376 | Burton, G., pers. comm. |
| Eu | UK | Hayle Estuary | 5931 | 8185 | 5760 |  |  |  | 350 | 6000 | 6000 |  | 5371 | Burton, G., pers. comm. |
| Eu | UK | Hodbarrow |  |  |  |  |  |  |  | 23000 | 23000 |  | 23000 | Burton, G., pers. comm. |
| Eu | UK | Ken-Dee Marshes | 1964 | 3815 | 2035 |  | 1000 |  |  | 10000 | 10000 |  | 4802 | Burton, G., pers. comm. |
| Eu | UK | Leighton Moss | 92531 | 85546 | 61891 | 33635 | 105341 |  | 119602 | 54929 | 76400 |  | 78734 | Burton, G., pers. comm. |
| Eu | UK | Loch Gruinart | 5261 | 4317 | 2593 | 977 | 155 |  | 8060 | 5743 | 11000 |  | 4763 | Burton, G., pers. comm. |
| Eu | UK | Loch Lomond and the Trossachs National Park |  |  |  | 1800000 | 2006000 | 2180000 |  |  |  |  | 1995333 | http://www.lochlomond-trossachs.org/images/stories/archive/file/stateofthepark/visitor_numbers.pdf (accessed 17/11/2014) |
| Eu | UK | Lochwinnoch | 21825 | 25948 | 25431 | 5755 | 34883 |  | 40781 | 40336 | 34110 |  | 28634 | Burton, G., pers. comm. |
| Eu | UK | Lodge | 38275 | 39603 | 36315 | 40450 | 47094 |  | 60674 | 51461 | 45575 |  | 44931 | Burton, G., pers. comm. |
| Eu | UK | Marshside | 11307 | 11290 | 4493 | 6991 | 13099 |  | 12693 | 25000 | 25000 |  | 13734 | Burton, G., pers. comm. |
| Eu | UK | Mersehead |  |  |  |  |  |  | 24620 | 27010 | 27110 |  | 26247 | Burton, G., pers. comm. |
| Eu | UK | Mid Yare Valley | 9134 | 10433 | 4612 | 3790 | 4263 |  | 10452 | 10876 | 13077 |  | 8330 | Burton, G., pers. comm. |
| Eu | UK | Minsmere | 68247 | 69371 | 57341 | 64300 | 69625 |  | 88222 | 91944 | 109790 |  | 77355 | Burton, G., pers. comm. |
| Eu | UK | Nagshead | 13000 | 13860 | 12594 | 2800 |  |  | 18000 | 18000 | 18000 |  | 13751 | Burton, G., pers. comm. |
| Eu | UK | Nene Washes | 548 | 730 |  | 255 | 703 |  |  |  |  |  | 559 | Burton, G., pers. comm. |
| Eu | UK | New Forest |  |  |  |  |  |  |  | 4300000 |  |  | 4300000 | [57] |
| Eu | UK | North Warren | 7100 | 4100 |  | 1800 | 5700 |  | 22400 | 22400 | 22400 |  | 12271 | Burton, G., pers. comm. |
| Eu | UK | North York Moors National Park |  |  |  |  |  |  |  | 7300000 |  |  | 7300000 | [57] |
| Eu | UK | Northumberland |  |  |  |  |  |  |  | 1200000 |  |  | 1200000 | [57] |
| Eu | UK | Northward Hill | 289 | 479 | 126 |  |  |  |  | 20500 | 21275 |  | 8534 | Burton, G., pers. comm. |
| Eu | UK | Old Moor |  |  |  |  |  |  | 41960 | 50245 | 79921 |  | 57375 | Burton, G., pers. comm. |
| Eu | UK | Otmoor |  |  |  |  |  |  |  | 3656 | 5000 |  | 4328 | Burton, G., pers. comm. |
| Eu | UK | Ouse Washes |  |  |  |  |  |  | 11634 | 10365 | 10271 |  | 10757 | Burton, G., pers. comm. |
| Eu | UK | Pulborough Brooks | 28067 | 79709 | 17585 | 22049 | 29179 |  | 121956 | 90000 | 97323 |  | 60734 | Burton, G., pers. comm. |
| Eu | UK | Radipole Lake | 35097 | 29417 | 25940 | 23985 | 26804 |  | 16048 | 25000 | 26020 |  | 26039 | Burton, G., pers. comm. |
| Eu | UK | Rye Meads | 11084 | 12289 | 8985 | 5080 | 12465 |  | 22412 | 15076 | 17259 |  | 13081 | Burton, G., pers. comm. |
| Eu | UK | Sandwell Valley | 24422 | 15163 | 14637 | 11165 | 14709 |  | 19665 | 33551 | 22538 |  | 19481 | Burton, G., pers. comm. |
| Eu | UK | Snettisham |  |  |  |  |  |  | 18716 | 17814 | 25999 |  | 20843 | Burton, G., pers. comm. |
| Eu | UK | The Broads |  |  |  |  |  |  |  | 700000 |  |  | 700000 | [57] |
| Eu | UK | The Lake District |  |  |  |  |  |  |  | 10500000 |  |  | 10500000 | [57] |
| Eu | UK | The Peak District |  |  |  |  |  |  |  | 10100000 |  |  | 10100000 | [57] |
| Eu | UK | The Yorkshire Dales |  |  |  |  |  |  |  | 3000000 |  |  | 3000000 | [57] |
| Eu | UK | Titchwell Marsh | 137700 | 136730 | 114414 | 111005 | 95690 |  | 111332 | 91166 | 86974 |  | 110626 | Burton, G., pers. comm. |
| Eu | UK | Tudeley Woods |  |  |  |  |  |  |  | 8000 | 8000 |  | 8000 | Burton, G., pers. comm. |
| Eu | UK | West Sedgemoor | 9482 | 10300 | 6800 | 3700 | 8200 |  | 11925 | 13935 | 15065 |  | 9926 | Burton, G., pers. comm. |
| Eu | UK | Wolves Wood | 3920 | 3650 |  |  | 4330 |  |  | 4000 | 4000 |  | 3980 | Burton, G., pers. comm. |
| Eu | UK | Wood of Cree | 7936 | 5816 | 8792 | 5814 | 3950 |  |  | 8000 | 9288 |  | 7085 | Burton, G., pers. comm. |
| LAm | Argentina | Ischigualasto - Talampaya Natural Park | 34000 |  |  |  |  |  |  |  |  |  | 34000 | [58] |
| LAm | Belize | Blue Hole National Park | 7098 | 6162 |  |  |  |  |  |  |  |  | 6630 | [59] |
| LAm | Belize | Cockscomb Basin Wildlife Sanctuary | 4078 | 3603 |  |  |  |  |  |  |  |  | 3841 | [59] |
| LAm | Belize | Community Baboon Sanctuary | 4676 | 4011 |  |  |  |  |  |  |  | 15000 | 7896 | [59]; [60] |
| LAm | Belize | Crooked Tree Wildlife Sanctuary | 1483 | 1619 |  |  |  |  |  |  |  |  | 1551 | [59] |
| LAm | Belize | Guanacaste National Park | 2567 | 2788 |  |  |  |  |  |  |  |  | 2678 | [59] |
| LAm | Belize | Mountain Pine Ridge Forest Reserve | 17896 | 25835 |  |  |  |  |  |  |  |  | 21866 | [59] |
| LAm | Bolivia | Apolobamba Integrated Management Area |  |  |  |  |  |  |  |  |  | 300 | 300 | Craigie, I., pers. comm. |
| LAm | Bolivia | Eduardo Avaroa Reserve |  | 17000 | 26000 | 31000 | 40000 | 43000 |  |  |  | 67000 | 37333 | [61]; http://en.wikipedia.org/wiki/Eduardo_Avaroa_Andean_Fauna_ National_Reserve (accessed 17/11/2014) |
| LAm | Bolivia | Madidi National Park |  |  |  |  |  |  |  | 7000 |  |  | 7000 | http://news.bbc.co.uk/1/hi/world/americas/4180442.stm (accessed 17/11/2014) |
| LAm | Bolivia | Noel Kempff Mercado National Park | 160 | 160 |  |  |  |  |  |  |  | 500 | 273 | [61]; Craigie, I., pers. comm. |
| LAm | Brazil | Aparados da Serra |  |  | 30600 | 32085 | 37844 | 56478 | 48503 | 49970 |  |  | 42580 | http://www.birdlist.org/downloads/parks/visitation_protected_areas_megacountries_with_megadiversity.pdf (accessed 17/11/2014) |
| LAm | Brazil | Brasilia |  |  | 182744 | 218124 | 256634 | 203150 | 195265 | 281016 |  |  | 222822 | http://www.birdlist.org/downloads/parks/visitation_protected_areas_megacountries_with_megadiversity.pdf (accessed 17/11/2014) |
| LAm | Brazil | Caparao |  |  | 25744 | 29642 | 25566 | 30459 | 30036 | 27391 |  |  | 28140 | http://www.birdlist.org/downloads/parks/visitation_protected_areas_megacountries_with_megadiversity.pdf (accessed 17/11/2014) |
| LAm | Brazil | Chapada does Veadeiros |  |  | 8027 | 16480 | 22158 | 24598 | 19506 | 14589 |  |  | 17560 | http://www.birdlist.org/downloads/parks/visitation_protected_areas_megacountries_with_megadiversity.pdf (accessed 17/11/2014) |
| LAm | Brazil | Chapada dos Guimaraes |  |  | 0 | 0 | 0 | 0 | 0 | 40869 |  |  | 6812 | http://www.birdlist.org/downloads/parks/visitation_protected_areas_megacountries_with_megadiversity.pdf (accessed 17/11/2014) |
| LAm | Brazil | Emas National Park |  |  | 0 | 0 | 0 | 0 | 0 | 1658 |  | 4000 | 808 | Craigie, I., pers. comm.; http://www.birdlist.org/downloads/parks/visitation_protected_areas_megacountries_with_megadiversity.pdf (accessed 17/11/2014) |
| LAm | Brazil | Guartelá State Park |  | 18137 |  |  |  |  |  |  |  |  | 18137 | http://www.pr.gov.br/turismo/areas_prioritarias.pdf (accessed 01/2008) |
| LAm | Brazil | Itatiaia |  |  | 97001 | 127713 | 119735 | 125633 | 126940 | 78002 |  |  | 112504 | http://www.birdlist.org/downloads/parks/visitation_protected_areas_megacountries_with_megadiversity.pdf (accessed 17/11/2014) |
| LAm | Brazil | Jaú National Park | 885 |  |  |  |  |  |  |  |  |  | 885 | [62] |
| LAm | Brazil | Monte Pascoal |  |  | 3460 | 2156 | 1139 | 1422 | 1048 | 1252 |  |  | 1746 | http://www.birdlist.org/downloads/parks/visitation_protected_areas_megacountries_with_megadiversity.pdf (accessed 17/11/2014) |
| LAm | Brazil | Parque Nacional da Serra do Divisor |  |  |  |  |  |  |  |  |  | 100 | 100 | Craigie, I., pers. comm. |
| LAm | Brazil | Serra da Bocaina |  |  | 8454 | 8672 | 6232 | 6728 | 5838 | 3876 |  |  | 6633 | http://www.birdlist.org/downloads/parks/visitation_protected_areas_megacountries_with_megadiversity.pdf (accessed 17/11/2014) |
| LAm | Brazil | Serra da Canastra |  |  | 20030 | 26502 | 30262 | 35412 | 27460 | 27670 |  |  | 27889 | http://www.birdlist.org/downloads/parks/visitation_protected_areas_megacountries_with_megadiversity.pdf (accessed 17/11/2014) |
| LAm | Brazil | Serra da Capivara |  |  | 0 | 5274 | 3014 | 3908 | 7107 | 6978 |  |  | 4380 | http://www.birdlist.org/downloads/parks/visitation_protected_areas_megacountries_with_megadiversity.pdf (accessed 17/11/2014) |
| LAm | Brazil | Serra das Confusoes |  |  | 0 | 0 | 0 | 0 | 0 | 1104 |  |  | 184 | http://www.birdlist.org/downloads/parks/visitation_protected_areas_megacountries_with_megadiversity.pdf (accessed 17/11/2014) |
| LAm | Brazil | Serra do Cipo |  |  | 432 | 0 | 11918 | 14342 | 13132 | 12900 |  |  | 8787 | http://www.birdlist.org/downloads/parks/visitation_protected_areas_megacountries_with_megadiversity.pdf (accessed 17/11/2014) |
| LAm | Brazil | Serra dos Orgaos |  |  | 28674 | 49912 | 53845 | 59994 | 46689 | 75076 |  |  | 52365 | http://www.birdlist.org/downloads/parks/visitation_protected_areas_megacountries_with_megadiversity.pdf (accessed 17/11/2014) |
| LAm | Brazil | Serra Geral |  |  | 14989 | 16629 | 17832 | 32551 | 31112 | 12628 |  |  | 20957 | http://www.birdlist.org/downloads/parks/visitation_protected_areas_megacountries_with_megadiversity.pdf (accessed 17/11/2014) |
| LAm | Brazil | Sete Cidades |  |  | 17641 | 16930 | 21566 | 21212 | 17951 | 17062 |  |  | 18727 | http://www.birdlist.org/downloads/parks/visitation_protected_areas_megacountries_with_megadiversity.pdf (accessed 17/11/2014) |
| LAm | Brazil | Tijuca |  |  | 622038 | 635498 | 497808 | 584192 | 1013631 | 1069066 |  |  | 737039 | http://www.birdlist.org/downloads/parks/visitation_protected_areas_megacountries_with_megadiversity.pdf (accessed 17/11/2014) |
| LAm | Brazil | Ubajara |  |  | 44787 | 43046 | 41867 | 48297 | 47852 | 42831 |  |  | 44780 | http://www.birdlist.org/downloads/parks/visitation_protected_areas_megacountries_with_megadiversity.pdf (accessed 17/11/2014) |
| LAm | Brazil | Vila Velha State Park |  | 125801 |  |  |  |  |  |  |  |  | 125801 | http://www.pr.gov.br/turismo/areas_prioritarias.pdf (accessed 01/2008) |
| LAm | Chile | Alacalufes |  |  |  |  |  |  |  | 0 |  |  | 0 | [63-69] |
| LAm | Chile | Alberto de Agostini |  |  |  |  |  |  |  | 0 |  |  | 0 | [63-69] |
| LAm | Chile | Alerce Andino |  | 7188 | 5890 | 5717 | 5544 | 5346 | 7132 | 6749 |  |  | 6224 | [63-69] |
| LAm | Chile | Alerce Costero |  | 588 | 633 | 646 | 419 | 436 | 392 | 575 |  |  | 527 | [63-69] |
| LAm | Chile | Alto Bio Bio |  | 13 | 16 | 45 | 37 | 125 | 129 | 69 |  |  | 62 | [63-69] |
| LAm | Chile | Altos de Lircay |  | 2232 | 4258 | 4793 | 3994 | 5272 | 4490 | 3609 |  |  | 4093 | [63-69] |
| LAm | Chile | Bernardo O'Higgins National Reserve |  |  |  |  |  | 11173 | 14334 | 15684 |  |  | 13730 | [63-69] |
| LAm | Chile | Bosque de Fray Jorge |  | 11625 | 10893 | 14241 | 12303 | 14034 |  | 13982 |  |  | 12846 | [63-69] |
| LAm | Chile | Cabo de Hornos |  |  |  |  |  |  |  | 0 |  |  | 0 | [63-69] |
| LAm | Chile | Cerro Castillo |  | 152 | 182 | 336 | 390 | 565 | 980 | 716 |  |  | 474 | [63-69] |
| LAm | Chile | Cerro Nielol |  | 52344 | 46832 | 38401 | 40310 | 42881 | 47496 | 44774 |  |  | 44720 | [63-69] |
| LAm | Chile | Chiloe |  | 7433 | 7186 | 8566 | 8536 | 9774 | 9289 | 9194 |  |  | 8568 | [63-69] |
| LAm | Chile | Coihaique |  | 5357 | 5379 | 4009 | 4158 | 4231 | 4693 | 3741 |  |  | 4510 | [63-69] |
| LAm | Chile | Conguillio |  | 33020 | 10802 | 9543 | 6877 | 23827 |  | 30152 |  |  | 19037 | [63-69] |
| LAm | Chile | Contulmo |  | 250 | 798 | 800 | 230 | 271 | 269 | 662 |  |  | 469 | [63-69] |
| LAm | Chile | Cueva del Milodon |  |  | 41939 | 43598 | 43598 | 45946 | 51195 | 52772 |  |  | 46508 | [63-69] |
| LAm | Chile | Dos Lagunas |  | 699 | 803 | 357 | 783 | 923 | 933 | 795 |  |  | 756 | [63-69] |
| LAm | Chile | El Morado |  | 7808 | 8475 | 7651 | 8155 | 9715 | 9158 | 9191 |  |  | 8593 | [63-69] |
| LAm | Chile | El Yali |  |  | 478 | 618 | 523 | 607 | 927 | 676 |  |  | 638 | [63-69] |
| LAm | Chile | Federico Albert |  | 15152 | 10795 | 13932 | 12313 | 13846 |  | 15434 |  |  | 13579 | [63-69] |
| LAm | Chile | Hornopiren |  |  |  |  |  |  |  | 0 |  |  | 0 | [63-69] |
| LAm | Chile | Huemules de Niblito |  |  |  |  |  |  | 8 | 0 |  |  | 4 | [63-69] |
| LAm | Chile | Huerquehue |  | 9421 | 10823 | 12330 | 12169 | 13340 | 15923 | 17904 |  |  | 13130 | [63-69] |
| LAm | Chile | Isla Guamblin |  |  |  |  |  |  |  | 0 |  |  | 0 | [63-69] |
| LAm | Chile | Isla Magdalena |  |  |  |  |  |  |  | 0 |  |  | 0 | [63-69] |
| LAm | Chile | Isla Mocha |  | 148 | 112 | 67 | 117 | 136 | 329 | 421 |  |  | 190 | [63-69] |
| LAm | Chile | Katalalixar |  |  |  |  |  |  |  | 0 |  |  | 0 | [63-69] |
| LAm | Chile | La Campana |  | 28510 | 33066 | 28558 | 27702 | 38723 |  | 35469 |  |  | 32005 | [63-69] |
| LAm | Chile | La Chimba |  |  |  |  |  |  |  | 0 |  |  | 0 | [63-69] |
| LAm | Chile | Lago Carlota |  |  |  |  |  |  |  | 0 |  |  | 0 | [63-69] |
| LAm | Chile | Lago Cochrane |  | 522 | 520 | 654 | 221 | 373 | 602 | 1274 |  |  | 595 | [63-69] |
| LAm | Chile | Lago Jeinimeni |  | 286 | 526 | 558 | 409 | 350 | 498 | 386 |  |  | 430 | [63-69] |
| LAm | Chile | Lago Las Torres |  | 425 | 1043 | 167 | 143 | 2 | 97 | 0 |  |  | 268 | [63-69] |
| LAm | Chile | Lago Palena |  |  |  |  |  |  |  | 0 |  |  | 0 | [63-69] |
| LAm | Chile | Lago Penuelas |  | 69011 | 34386 | 43164 | 28926 | 38874 | 27634 | 25920 |  |  | 38274 | [63-69] |
| LAm | Chile | Lago Rosselot |  |  |  |  |  |  |  | 0 |  |  | 0 | [63-69] |
| LAm | Chile | Laguna de Los Cisnes |  |  |  |  |  |  |  | 0 |  |  | 0 | [63-69] |
| LAm | Chile | Laguna de Torca |  | 4895 | 3704 | 5373 | 4165 | 4986 | 6202 | 6116 |  |  | 5063 | [63-69] |
| LAm | Chile | Laguna del Laja |  | 13172 | 11184 | 7414 | 14725 | 14000 |  | 14829 |  |  | 12554 | [63-69] |
| LAm | Chile | Laguna Parrillar |  |  | 2717 | 2913 | 2913 | 4398 | 5251 | 5440 |  |  | 3939 | [63-69] |
| LAm | Chile | Laguna San Rafael |  | 488 | 416 | 513 | 646 | 564 | 569 | 483 |  |  | 526 | [63-69] |
| LAm | Chile | Las Chinchillas |  | 981 | 1728 | 1627 | 1720 | 1810 | 3273 | 4049 |  |  | 2170 | [63-69] |
| LAm | Chile | Las Guaitecas |  |  |  |  |  |  |  | 0 |  |  | 0 | [63-69] |
| LAm | Chile | Las Palmas de Cocalan |  |  |  |  |  |  |  | 0 |  |  | 0 | [63-69] |
| LAm | Chile | Las Vicuñas |  | 545 | 19 | 584 | 592 | 512 | 624 | 435 |  |  | 473 | [63-69] |
| LAm | Chile | Lauca |  | 19654 | 17224 | 16293 | 14410 | 11170 | 14445 | 14318 |  |  | 15359 | [63-69] |
| LAm | Chile | Llanos de Challe |  | 633 | 947 | 409 | 2034 | 576 | 1937 | 1427 |  |  | 1138 | [63-69] |
| LAm | Chile | Llanquihue |  | 1228 | 1568 | 1417 | 1420 | 2085 | 1990 | 2264 |  |  | 1710 | [63-69] |
| LAm | Chile | Llullaillaco |  |  |  |  |  |  |  | 0 |  |  | 0 | [63-69] |
| LAm | Chile | Los Bellotos del Melado |  |  |  |  | 5 | 116 | 462 | 387 |  |  | 243 | [63-69] |
| LAm | Chile | Los Cipreses |  | 4454 | 4283 | 3200 | 5029 | 6835 |  | 5906 |  |  | 4951 | [63-69] |
| LAm | Chile | Los Flamencos |  | 24914 | 23070 | 16249 | 33398 | 55867 | 123959 | 136009 |  |  | 59067 | [63-69] |
| LAm | Chile | Los Queules |  | 114 |  |  | 15 | 149 | 144 | 118 |  |  | 108 | [63-69] |
| LAm | Chile | Los Ruiles |  | 4449 |  | 5185 | 4383 | 3766 | 3041 | 2673 |  |  | 3916 | [63-69] |
| LAm | Chile | Magallanes |  |  | 2802 | 3442 | 3442 | 8193 | 10170 | 9706 |  |  | 6293 | [63-69] |
| LAm | Chile | Malalcahuello |  | 9299 | 1518 | 762 | 2781 | 6936 | 26375 | 18880 |  |  | 9507 | [63-69] |
| LAm | Chile | Malleco |  | 120 | 53 |  |  |  | 84 | 101 |  |  | 90 | [63-69] |
| LAm | Chile | Mocho Choshuenco |  |  |  |  |  |  |  | 0 |  |  | 0 | [63-69] |
| LAm | Chile | Nahuelbuta |  | 5002 | 7135 | 6264 | 5289 | 7198 | 6502 | 6023 |  |  | 6202 | [63-69] |
| LAm | Chile | Nalca |  |  |  |  |  |  |  | 0 |  |  | 0 | [63-69] |
| LAm | Chile | National Park Villarrica |  | 8827 | 21854 | 21041 | 20113 | 25229 | 58206 | 54251 |  |  | 29932 | [63-69] |
| LAm | Chile | National Reserve Villarrica |  |  |  |  |  |  |  | 0 |  |  | 0 | [63-69] |
| LAm | Chile | Nevado de tres Cruces |  | 441 | 431 | 569 | 455 | 366 | 308 | 244 |  |  | 402 | [63-69] |
| LAm | Chile | Ñuble |  | 236 | 286 | 283 | 254 | 518 | 501 | 571 |  |  | 378 | [63-69] |
| LAm | Chile | Pali Aiki |  | 688 | 536 | 1130 | 1130 | 1159 | 1158 | 1418 |  |  | 1031 | [63-69] |
| LAm | Chile | Pampa del tamarugal |  | 4615 | 6976 | 7302 | 7081 | 8529 | 6937 | 9471 |  |  | 7273 | [63-69] |
| LAm | Chile | Pan de Azñcar |  | 16137 | 14412 | 10559 | 10869 | 9164 |  | 12568 |  |  | 12285 | [63-69] |
| LAm | Chile | Pichasca |  | 1413 | 1516 | 5486 | 4746 | 3768 | 3756 | 3986 |  |  | 3524 | [63-69] |
| LAm | Chile | Puyehue |  | 98198 | 290699 | 359178 | 312907 | 337035 | 351630 | 380054 |  |  | 304243 | [63-69] |
| LAm | Chile | Queulat |  | 5262 | 7287 | 3976 | 6195 | 5930 | 6195 | 7902 |  |  | 6107 | [63-69] |
| LAm | Chile | Radal Siete Tazas |  | 13292 | 20869 | 21916 | 17552 | 22835 | 21534 | 20995 |  |  | 19856 | [63-69] |
| LAm | Chile | Ralco |  | 307 | 361 | 294 | 109 | 492 | 434 | 394 |  |  | 342 | [63-69] |
| LAm | Chile | Rio Blanco |  |  |  |  |  |  |  | 0 |  |  | 0 | [63-69] |
| LAm | Chile | Rio Clarillo |  | 36056 | 40169 | 27531 | 32228 | 39957 | 34452 | 46347 |  |  | 36677 | [63-69] |
| LAm | Chile | Rio Simpson |  | 5833 | 4524 | 6261 | 5171 | 6155 | 6793 | 6818 |  |  | 5936 | [63-69] |
| LAm | Chile | Robleria del Cobre de Loncha |  |  |  |  |  |  |  | 2094 |  |  | 2094 | [63-69] |
| LAm | Chile | Salar de surire |  | 644 | 450 | 761 | 910 | 819 | 678 | 435 |  |  | 671 | [63-69] |
| LAm | Chile | Tolhuaca |  | 1353 | 1260 | 1350 | 508 | 1623 | 1304 | 1335 |  |  | 1248 | [63-69] |
| LAm | Chile | Torres del Paine |  | 63806 | 71092 | 75598 | 75598 | 86467 | 98065 | 107091 | 114373 | 128402 | 91166 | [63-69]; [http://en.mercopress.com/2008/01/29/record-visitors-to-torres-del-paine-park-in-south-chile](file:///C:\Users\apb12\AppData\Local\Temp\http#://en.mercopress.com/2008/01/29/record-visitors-to-torres-del-paine-park-in-south-chile) (accessed 18/12/2014) |
| LAm | Chile | Trapananda |  |  |  |  |  |  |  | 0 |  |  | 0 | [63-69] |
| LAm | Chile | Valdivia |  |  |  |  | 103 |  |  | 0 |  |  | 52 | [63-69] |
| LAm | Chile | Vicente Perez Rosales |  | 171179 | 171548 | 154031 | 147919 | 153217 | 176370 | 182515 |  |  | 165254 | [63-69] |
| LAm | Chile | Volcan Isluga |  | 160 | 100 | 475 | 739 | 114 | 140 | 89 |  |  | 260 | [63-69] |
| LAm | Peru | Ampay National Sanctuary |  |  |  |  |  |  | 5215 |  |  |  | 5215 | Kirkby, C., pers. comm. |
| LAm | Peru | Bahuaja Sonene National Park |  |  |  |  |  |  |  |  |  | 80 | 80 | Craigie, I., pers. comm. |
| LAm | Peru | Historic Sanctuary of Machu Picchu |  |  |  |  |  |  |  |  | 691623 |  | 691623 | [70] |
| LAm | Peru | Huascarán National Park |  |  |  |  |  |  | 109302 |  |  |  | 109302 | Kirkby, C., pers. comm. |
| LAm | Peru | Huascarán WHS |  |  | 109063 |  |  |  |  |  |  |  | 109063 | http://www.parkswatch.org/parkprofiles/pdf/hunp_eng.pdf (accessed 17/11/2014) |
| LAm | Peru | Huayllay National Sanctuary |  |  |  |  |  |  | 18414 |  |  |  | 18414 | Kirkby, C., pers. comm. |
| LAm | Peru | Junín National Reserve |  |  |  |  |  |  | 250 |  |  |  | 250 | Kirkby, C., pers. comm. |
| LAm | Peru | Lachay National Reserve |  |  |  |  |  |  | 23593 |  |  |  | 23593 | Kirkby, C., pers. comm. |
| LAm | Peru | Lagunas de Mejía National Sanctuary |  |  |  |  |  |  | 1393 |  |  |  | 1393 | Kirkby, C., pers. comm. |
| LAm | Peru | Pacaya Samiria National Reserve |  |  |  |  |  |  | 1821 |  |  |  | 1821 | Kirkby, C., pers. comm. |
| LAm | Peru | Parque Nacional Cerros de Amotape |  |  |  |  |  |  |  |  |  | 2151 | 2151 | Craigie, I., pers. comm. |
| LAm | Peru | Tambopata National Reserve |  |  |  |  |  |  | 13982 |  |  |  | 13982 | Kirkby, C., pers. comm. |
| LAm | Peru | Tingo María National Park |  |  |  |  |  |  | 23419 |  |  |  | 23419 | Kirkby, C., pers. comm. |
| LAm | Saint Lucia | Pitons Management Area |  |  |  |  |  | 200000 |  |  |  |  | 200000 | [33] |
| LAm | Venezuela | Canaima National Park | 100000 |  |  |  |  |  |  |  |  |  | 100000 | [33] |
| LAm | Venezuela | Los Roques Archipelago National Park |  |  |  | 75000 |  |  |  |  |  |  | 75000 | http://www.parkswatch.org/parkprofiles/pdf/ronp_eng.pdf (accessed 17/11/2014) |
| NAm | Canada | Banff National Park |  |  |  | 3106470 | 3088390 | 2935140 | 3139934 | 3174043 | 3297460 | 3348632 | 3155724 | http://www.pc.gc.ca/docs/pc/attend/index_e.asp (accessed 17/11/2014) |
| NAm | Canada | Cape Breton Highlands National Park |  |  |  | 366617 | 404239 | 396033 | 322259 | 311229 | 340207 | 298131 | 348388 | http://www.pc.gc.ca/docs/pc/attend/index_e.asp (accessed 17/11/2014) |
| NAm | Canada | Dinosaur Provincial Park |  |  | 84340 |  |  |  |  |  |  |  | 84340 | http://www.pc.gc.ca/eng/docs/pm-wh/rspm-whsr/rapports-reports/r2.aspx (accessed 17/11/2014) |
| NAm | Canada | Elk Island National Park |  |  |  | 209895 | 190272 | 189418 | 171447 | 180946 | 182736 | 186855 | 187367 | http://www.pc.gc.ca/docs/pc/attend/index_e.asp (accessed 17/11/2014) |
| NAm | Canada | Fundy National Park |  |  |  | 320414 | 335058 | 316437 | 288579 | 285953 | 258876 | 249860 | 293597 | http://www.pc.gc.ca/docs/pc/attend/index_e.asp (accessed 17/11/2014) |
| NAm | Canada | Grasslands National Park |  |  |  | 6773 | 7043 | 4927 | 6642 | 6506 | 6552 | 5814 | 6322 | http://www.pc.gc.ca/docs/pc/attend/index_e.asp (accessed 17/11/2014) |
| NAm | Canada | Gros Morne National Park |  |  |  | 118071 | 118071 | 118071 | 157830 | 156020 | 156776 | 159138 | 140568 | http://www.pc.gc.ca/docs/pc/attend/index_e.asp (accessed 17/11/2014) |
| NAm | Canada | Gwaii Haanas National Park |  |  |  | 1925 | 1993 | 2187 | 2056 | 1868 | 1868 | 2098 | 1999 | http://www.pc.gc.ca/docs/pc/attend/index_e.asp (accessed 17/11/2014) |
| NAm | Canada | Jasper National Park |  |  |  | 1846506 | 1838175 | 1686756 | 1880153 | 1906928 | 1991615 | 2054877 | 1886430 | http://www.pc.gc.ca/docs/pc/attend/index_e.asp (accessed 17/11/2014) |
| NAm | Canada | Kejimkujik National Park |  |  |  | 66472 | 61668 | 56763 | 48929 | 46848 | 40813 | 43903 | 52199 | http://www.pc.gc.ca/docs/pc/attend/index_e.asp (accessed 17/11/2014) |
| NAm | Canada | Kluane National Park |  |  |  | 48385 | 47512 | 39075 | 45491 | 44277 | 41582 | 40510 | 43833 | http://www.pc.gc.ca/docs/pc/attend/index_e.asp (accessed 17/11/2014) |
| NAm | Canada | Kootenay National Park |  |  |  | 17315 | 417830 | 390714 | 420721 | 425006 | 445364 | 454520 | 367353 | http://www.pc.gc.ca/docs/pc/attend/index_e.asp (accessed 17/11/2014) |
| NAm | Canada | Mauricie National Park |  |  |  | 96786 | 188389 | 173075 | 184083 | 163306 | 151223 | 149521 | 158055 | http://www.pc.gc.ca/docs/pc/attend/index_e.asp (accessed 17/11/2014) |
| NAm | Canada | Nahanni National Park |  |  |  | 936 | 977 | 1015 | 885 | 1021 | 796 | 956 | 941 | http://www.pc.gc.ca/docs/pc/attend/index_e.asp (accessed 17/11/2014) |
| NAm | Canada | Point Pelee National Park |  |  |  | 331193 | 306477 | 285930 | 272006 | 230222 | 226902 | 228815 | 268792 | http://www.pc.gc.ca/docs/pc/attend/index_e.asp (accessed 17/11/2014) |
| NAm | Canada | Prince Albert National Park |  |  |  | 230530 | 223915 | 239918 | 221006 | 216179 | 219372 | 222498 | 224774 | http://www.pc.gc.ca/docs/pc/attend/index_e.asp (accessed 17/11/2014) |
| NAm | Canada | Pukaskwa National Park |  |  |  | 8488 | 9406 | 8263 | 7250 | 8170 | 6689 | 7737 | 8000 | http://www.pc.gc.ca/docs/pc/attend/index_e.asp (accessed 17/11/2014) |
| NAm | Canada | Riding Mountain, Manitoba |  |  |  | 282759 | 246387 | 247563 | 245565 | 249493 | 249493 | 249493 | 252965 | http://www.pc.gc.ca/docs/pc/attend/index_e.asp (accessed 17/11/2014) |
| NAm | Canada | Tombstone Territorial Park |  | 253 | 213 | 155 | 223 | 215 | 166 | 912 | 1143 |  | 410 | Jones, A., pers. comm. |
| NAm | Canada | Waterton Lakes National Park |  |  |  | 414121 | 390825 | 370939 | 366431 | 363043 | 364042 | 386524 | 379418 | http://www.pc.gc.ca/docs/pc/attend/index_e.asp (accessed 17/11/2014) |
| NAm | Canada | Yoho National Park |  |  |  | 568479 | 563115 | 501270 | 576413 | 581309 | 609395 | 606937 | 572417 | http://www.pc.gc.ca/docs/pc/attend/index_e.asp (accessed 17/11/2014) |
| NAm | USA | Agate Fossil Beds NM |  |  |  |  |  | 16233 | 15235 | 14209 | 13521 | 12997 | 14439 | https://irma.nps.gov/Stats/ (accessed 17/11/2014) |
| NAm | USA | Aniakchak NM & PRES |  |  |  |  |  | 154 | 285 | 285 | 60 | 26 | 162 | https://irma.nps.gov/Stats/ (accessed 17/11/2014) |
| NAm | USA | Arches National Park | 837161 | 869980 | 786429 | 754026 | 769672 | 757781 | 733131 | 781670 | 833049 | 860181 | 798308 | https://irma.nps.gov/Stats/ (accessed 17/11/2014) |
| NAm | USA | Assateague Island NS |  |  |  |  |  | 2020666 | 2048789 | 1996502 | 1932817 | 2110918 | 2021938 | https://irma.nps.gov/Stats/ (accessed 17/11/2014) |
| NAm | USA | Badlands National Park | 1021049 | 950453 | 1105824 | 955469 | 908898 | 871034 | 936030 | 909146 | 840118 | 886654 | 938468 | https://irma.nps.gov/Stats/ (accessed 17/11/2014) |
| NAm | USA | Bering Land Bridge NPRES |  |  |  |  |  | 2425 | 2710 | 2428 | 1265 | 796 | 1925 | https://irma.nps.gov/Stats/ (accessed 17/11/2014) |
| NAm | USA | Big Bend National Park | 338442 | 327649 | 262320 | 328927 | 327747 | 312384 | 357723 | 398583 | 298717 | 364856 | 331735 | https://irma.nps.gov/Stats/ (accessed 17/11/2014) |
| NAm | USA | Big Cypress NPRES |  |  |  |  |  | 400902 | 385194 | 768687 | 825857 | 822864 | 640701 | https://irma.nps.gov/Stats/ (accessed 17/11/2014) |
| NAm | USA | Black Canyon of the Gunnison NP |  |  |  |  |  | 167247 | 175581 | 180814 | 160450 | 219576 | 180734 | https://irma.nps.gov/Stats/ (accessed 17/11/2014) |
| NAm | USA | Bluestone NSR |  |  |  |  |  | 50302 | 39590 | 45146 | 46093 | 48061 | 45838 | https://irma.nps.gov/Stats/ (accessed 17/11/2014) |
| NAm | USA | Bryce Canyon National Park | 1166331 | 1081521 | 1099275 | 1068619 | 886436 | 903760 | 987253 | 1017681 | 890676 | 1012563 | 1011412 | https://irma.nps.gov/Stats/ (accessed 17/11/2014) |
| NAm | USA | Canaveral NS |  |  |  |  |  | 1045898 | 1050211 | 1007446 | 1005402 | 1038449 | 1029481 | https://irma.nps.gov/Stats/ (accessed 17/11/2014) |
| NAm | USA | Canyonlands National Park | 436524 | 446160 | 401558 | 368592 | 367078 | 386986 | 371706 | 393381 | 392537 | 417560 | 398208 | https://irma.nps.gov/Stats/ (accessed 17/11/2014) |
| NAm | USA | Cape Cod NS |  |  |  |  |  | 4066365 | 4106840 | 3712812 | 4487716 | 4351609 | 4145068 | https://irma.nps.gov/Stats/ (accessed 17/11/2014) |
| NAm | USA | Cape Hatteras NS |  |  |  |  |  | 2660535 | 2208189 | 2260628 | 2125005 | 2237378 | 2298347 | https://irma.nps.gov/Stats/ (accessed 17/11/2014) |
| NAm | USA | Cape Krusenstern NM |  |  |  |  |  | 3587 | 4330 | 4664 | 2598 | 745 | 3185 | https://irma.nps.gov/Stats/ (accessed 17/11/2014) |
| NAm | USA | Cape Lookout NS |  |  |  |  |  | 704480 | 720216 | 692857 | 803155 | 860602 | 756262 | https://irma.nps.gov/Stats/ (accessed 17/11/2014) |
| NAm | USA | Capitol Reef National Park | 656026 | 680153 | 612656 | 527760 | 525646 | 535441 | 549708 | 550255 | 511511 | 554907 | 570406 | https://irma.nps.gov/Stats/ (accessed 17/11/2014) |
| NAm | USA | Capulin Volcano NM |  |  |  |  |  | 61373 | 57692 | 53521 | 49823 | 49182 | 54318 | https://irma.nps.gov/Stats/ (accessed 17/11/2014) |
| NAm | USA | Carlsbad Caverns National Park | 522076 |  |  |  |  | 457631 | 419599 | 413786 | 407367 | 409560 | 438337 | www.nationalparked.com/US/Carlsbad_Caverns/Visitation_History.php (accessed 17/11/2014) |
| NAm | USA | Catoctin Mountain Park |  |  |  |  |  | 621114 | 699274 | 515193 | 526898 | 591191 | 590734 | https://irma.nps.gov/Stats/ (accessed 17/11/2014) |
| NAm | USA | Cedar Breaks NM |  |  |  |  |  | 569455 | 516331 | 505158 | 488376 | 514871 | 518838 | https://irma.nps.gov/Stats/ (accessed 17/11/2014) |
| NAm | USA | Chickasaw NRA |  |  |  |  |  | 1391334 | 1275733 | 1295212 | 1343793 | 1368806 | 1334976 | https://irma.nps.gov/Stats/ (accessed 17/11/2014) |
| NAm | USA | City of Rocks NRES |  |  |  |  |  | 79879 | 76586 | 67235 | 77131 | 79533 | 76073 | https://irma.nps.gov/Stats/ (accessed 17/11/2014) |
| NAm | USA | Colorado NM |  |  |  |  |  | 336642 | 352579 | 347136 | 332654 | 395260 | 352854 | https://irma.nps.gov/Stats/ (accessed 17/11/2014) |
| NAm | USA | Congaree swamp National Park | 93725 | 82223 | 95619 | 119738 | 101620 | 107114 | 126247 | 84301 | 134045 | 115524 | 106016 | https://irma.nps.gov/Stats/ (accessed 17/11/2014) |
| NAm | USA | Crater Lake National Park | 471865 | 417992 | 426883 | 457373 | 456620 | 479183 | 417066 | 447240 | 388972 | 468958 | 443215 | https://irma.nps.gov/Stats/ (accessed 17/11/2014) |
| NAm | USA | Craters of the Moon NM |  |  |  |  |  | 178824 | 183111 | 203332 | 176998 | 221672 | 192787 | https://irma.nps.gov/Stats/ (accessed 17/11/2014) |
| NAm | USA | Cumberland Island NS |  |  |  |  |  | 41727 | 38287 | 40291 | 44025 | 72449 | 47356 | https://irma.nps.gov/Stats/ (accessed 17/11/2014) |
| NAm | USA | Cuyahoga Valley National Park | 3467107 | 3324284 | 3324918 | 3123353 | 3217935 | 2879591 | 3306175 | 2533827 | 2468816 | 2486656 | 3013266 | https://irma.nps.gov/Stats/ (accessed 17/11/2014) |
| NAm | USA | Death Valley National Park | 1177746 | 1227583 | 1179094 | 1014636 | 897596 | 890375 | 764820 | 800113 | 744440 | 704122 | 940053 | https://irma.nps.gov/Stats/ (accessed 17/11/2014) |
| NAm | USA | Delaware Water Gap NRA |  |  |  |  |  | 5059410 | 5052264 | 5052264 | 5254216 | 4836229 | 5050877 | https://irma.nps.gov/Stats/ (accessed 17/11/2014) |
| NAm | USA | Denali National Park | 372519 | 386867 | 363983 | 360191 | 311335 | 360189 | 404236 | 403520 | 415935 | 458308 | 383708 | https://irma.nps.gov/Stats/ (accessed 17/11/2014) |
| NAm | USA | Devils Postpile NM |  |  |  |  |  | 118550 | 114788 | 67402 | 105303 | 120758 | 105360 | https://irma.nps.gov/Stats/ (accessed 17/11/2014) |
| NAm | USA | Devils Tower NM |  |  |  |  |  | 396266 | 386558 | 369575 | 335764 | 322272 | 362087 | https://irma.nps.gov/Stats/ (accessed 17/11/2014) |
| NAm | USA | El Malpais NM |  |  |  |  |  | 151127 | 137170 | 116194 | 107792 | 110639 | 124584 | https://irma.nps.gov/Stats/ (accessed 17/11/2014) |
| NAm | USA | El Morro NM |  |  |  |  |  | 57889 | 65877 | 51825 | 52297 | 51492 | 55876 | https://irma.nps.gov/Stats/ (accessed 17/11/2014) |
| NAm | USA | Everglades National Park | 1118215 | 1073982 | 995390 | 1049851 | 968909 | 1040648 | 1181355 | 1233837 | 954022 | 1074764 | 1069097 | https://irma.nps.gov/Stats/ (accessed 17/11/2014) |
| NAm | USA | Fire Island NS |  |  |  |  |  | 629858 | 819161 | 670456 | 636030 | 616233 | 674348 | https://irma.nps.gov/Stats/ (accessed 17/11/2014) |
| NAm | USA | Florissant Fossil Beds NM |  |  |  |  |  | 67537 | 61258 | 59508 | 56094 | 55992 | 60078 | https://irma.nps.gov/Stats/ (accessed 17/11/2014) |
| NAm | USA | Fossil Butte NM |  |  |  |  |  | 19417 | 18288 | 17779 | 16631 | 19853 | 18394 | https://irma.nps.gov/Stats/ (accessed 17/11/2014) |
| NAm | USA | Gates of the Arctic National Park | 8266 | 8166 | 11278 | 4505 | 6648 | 5075 | 10282 | 9459 | 9982 | 10942 | 8460 | https://irma.nps.gov/Stats/ (accessed 17/11/2014) |
| NAm | USA | Gauley River NRA |  |  |  |  |  | 148793 | 155183 | 128796 | 116854 | 118169 | 133559 | https://irma.nps.gov/Stats/ (accessed 17/11/2014) |
| NAm | USA | Glacier NP |  |  |  |  |  | 1664046 | 2033933 | 1925101 | 1964399 | 2083329 | 1934162 | https://irma.nps.gov/Stats/ (accessed 17/11/2014) |
| NAm | USA | Golden Gate NRA |  |  |  |  |  | 13854750 | 13270547 | 13602629 | 13486824 | 14397313 | 13722413 | https://irma.nps.gov/Stats/ (accessed 17/11/2014) |
| NAm | USA | Grand Canyon National Park | 4239682 | 4575124 | 4460228 | 4104809 | 4001974 | 4124900 | 4326234 | 4401522 | 4279439 | 4413668 | 4292758 | https://irma.nps.gov/Stats/ (accessed 17/11/2014) |
| NAm | USA | Grand Teton National Park | 2757060 | 2680025 | 2590624 | 2535108 | 2612629 | 2355693 | 2360373 | 2463442 | 2406476 | 2588574 | 2535000 | https://irma.nps.gov/Stats/ (accessed 17/11/2014) |
| NAm | USA | Great Basin National Park | 80363 | 90119 | 81045 | 81712 | 85777 | 87020 | 79879 | 77741 | 78524 | 81364 | 82354 | https://irma.nps.gov/Stats/ (accessed 17/11/2014) |
| NAm | USA | Great Sand Dunes NP & PRES |  |  |  |  |  | 251375 | 267204 | 279589 | 258660 | 285121 | 268390 | https://irma.nps.gov/Stats/ (accessed 17/11/2014) |
| NAm | USA | Guadalupe National Park | 227924 | 219591 | 198762 | 222307 | 202911 | 181357 | 182351 | 170383 | 174157 | 165110 | 194485 | https://irma.nps.gov/Stats/ (accessed 17/11/2014) |
| NAm | USA | Hagerman Fossil Beds NM |  |  |  |  |  | 15921 | 21801 | 23544 | 25875 | 24779 | 22384 | https://irma.nps.gov/Stats/ (accessed 17/11/2014) |
| NAm | USA | Haleakala National Park | 1476842 | 1963187 | 1620083 | 1410974 | 1521080 | 1444668 | 1455477 | 1474681 | 1426068 | 1322817 | 1511588 | https://irma.nps.gov/Stats/ (accessed 17/11/2014) |
| NAm | USA | Hawaii Volcanoes National Park | 1352373 | 1502855 | 1514636 | 1343286 | 1110998 | 1122524 | 1307391 | 1661196 | 1612246 | 1467779 | 1399528 | https://irma.nps.gov/Stats/ (accessed 17/11/2014) |
| NAm | USA | Indiana Dunes NL |  |  |  |  |  | 1953449 | 1810330 | 2127336 | 1938132 | 1972344 | 1960318 | https://irma.nps.gov/Stats/ (accessed 17/11/2014) |
| NAm | USA | Jewel Cave NM |  |  |  |  |  | 126363 | 108948 | 102318 | 97395 | 105321 | 108069 | https://irma.nps.gov/Stats/ (accessed 17/11/2014) |
| NAm | USA | John Day Fossil Beds NM |  |  |  |  |  | 108181 | 117613 | 124937 | 119656 | 124704 | 119018 | https://irma.nps.gov/Stats/ (accessed 17/11/2014) |
| NAm | USA | Joshua Tree National Park | 1410312 | 1316340 | 1233935 | 1280917 | 1178376 | 1283346 | 1243659 | 1375111 | 1256421 | 1298979 | 1287740 | https://irma.nps.gov/Stats/ (accessed 17/11/2014) |
| NAm | USA | Katmai NP & PRES |  |  |  |  |  | 51589 | 56787 | 54274 | 68630 | 82634 | 62783 | https://irma.nps.gov/Stats/ (accessed 17/11/2014) |
| NAm | USA | Kenai Fjords National Park | 263948 | 290673 | 254790 | 262353 | 251799 | 243719 | 244232 | 258297 | 251630 | 284604 | 260605 | https://irma.nps.gov/Stats/ (accessed 17/11/2014) |
| NAm | USA | Kings Canyon/Sequoia | 1401515 | 1432763 | 1367934 | 1412114 | 1465712 | 1535284 | 1525212 | 1599736 | 1507273 | 1559666 | 1480721 | https://irma.nps.gov/Stats/ (accessed 17/11/2014) |
| NAm | USA | Kobuk Valley National Park | 5550 | 6309 | 2646 | 4217 | 4046 | 4006 | 4744 | 5037 | 3005 | 847 | 4041 | https://irma.nps.gov/Stats/ (accessed 17/11/2014) |
| NAm | USA | Lake Chelan NRA |  |  |  |  |  | 35549 | 42529 | 29783 | 35151 | 34665 | 35535 | https://irma.nps.gov/Stats/ (accessed 17/11/2014) |
| NAm | USA | Lake Clark National Park | 11335 | 6190 | 6493 | 4397 | 4325 | 4505 | 4906 | 5408 |  |  | 5945 | https://irma.nps.gov/Stats/ (accessed 17/11/2014) |
| NAm | USA | Lake Mead NRA |  |  |  |  |  | 7915581 | 7819984 | 7692438 | 7777753 | 7622139 | 7765579 | https://irma.nps.gov/Stats/ (accessed 17/11/2014) |
| NAm | USA | Landels-Hill Big Creek Reserve | 918 | 1264 | 955 | 1053 | 1116 |  |  |  |  |  | 1061 | http://bigcreek.ucnrs.org/annual/user_statistics/visitor_statistics.html (accessed 17/11/2014) |
| NAm | USA | Lassen Volcanic National Park | 318049 | 353756 | 374911 | 376695 | 388149 | 404384 | 379667 | 365535 | 388741 | 395057 | 374494 | https://irma.nps.gov/Stats/ (accessed 17/11/2014) |
| NAm | USA | Lava Beds NM |  |  |  |  |  | 119515 | 106940 | 107475 | 104490 | 102629 | 108210 | https://irma.nps.gov/Stats/ (accessed 17/11/2014) |
| NAm | USA | Mammoth Cave National Park | 2113992 | 1710983 | 1749268 | 1883580 | 1891307 | 1869137 | 1888267 | 1878006 | 597934 | 487305 | 1606978 | https://irma.nps.gov/Stats/ (accessed 17/11/2014) |
| NAm | USA | Mesa Verde NP |  |  |  |  |  | 438590 | 446811 | 498333 | 557248 | 541102 | 496417 | https://irma.nps.gov/Stats/ (accessed 17/11/2014) |
| NAm | USA | Mount Rainier National Park | 1353793 | 1291397 | 1344833 | 1301103 | 1310390 | 1262351 | 1217750 | 1173897 | 1113601 | 1047685 | 1241680 | https://irma.nps.gov/Stats/ (accessed 17/11/2014) |
| NAm | USA | Muir Woods NM |  |  |  |  |  | 719350 | 778368 | 775941 | 741768 | 811033 | 765292 | https://irma.nps.gov/Stats/ (accessed 17/11/2014) |
| NAm | USA | Natural Bridges NM |  |  |  |  |  | 98874 | 96106 | 121247 | 91288 | 88316 | 99166 | https://irma.nps.gov/Stats/ (accessed 17/11/2014) |
| NAm | USA | North Cascades National Park | 32753 | 21488 | 25704 | 27739 | 20691 | 20724 | 16912 | 18686 | 19167 | 19534 | 22340 | https://irma.nps.gov/Stats/ (accessed 17/11/2014) |
| NAm | USA | Organ Pipe Cactus NM |  |  |  |  |  | 277566 | 229584 | 280068 | 313103 | 341594 | 288383 | https://irma.nps.gov/Stats/ (accessed 17/11/2014) |
| NAm | USA | Ozark NSR |  |  |  |  |  | 1301415 | 1317746 | 1554729 | 1491380 | 1789783 | 1491011 | https://irma.nps.gov/Stats/ (accessed 17/11/2014) |
| NAm | USA | Padre Island NS |  |  |  |  |  | 568737 | 643792 | 666580 | 730994 | 658317 | 653684 | https://irma.nps.gov/Stats/ (accessed 17/11/2014) |
| NAm | USA | Petrified Forest National Park | 816506 | 666978 | 605192 | 583904 | 571586 | 586245 | 580000 | 598378 | 581681 | 563590 | 615406 | https://irma.nps.gov/Stats/ (accessed 17/11/2014) |
| NAm | USA | Pictured Rocks NL |  |  |  |  |  | 382456 | 381854 | 476888 | 419298 | 441521 | 420403 | https://irma.nps.gov/Stats/ (accessed 17/11/2014) |
| NAm | USA | Pinnacles NM |  |  |  |  |  | 162791 | 157397 | 170994 | 158824 | 161019 | 162205 | https://irma.nps.gov/Stats/ (accessed 17/11/2014) |
| NAm | USA | Piscataway Park |  |  |  |  |  | 184823 | 190745 | 176174 | 199443 | 184636 | 187164 | https://irma.nps.gov/Stats/ (accessed 17/11/2014) |
| NAm | USA | Point Reyes NS |  |  |  |  |  | 2224882 | 1960055 | 1988585 | 2065083 | 2206294 | 2088980 | https://irma.nps.gov/Stats/ (accessed 17/11/2014) |
| NAm | USA | Rainbow Bridge NM |  |  |  |  |  | 98865 | 73675 | 81206 | 87642 | 81607 | 84599 | https://irma.nps.gov/Stats/ (accessed 17/11/2014) |
| NAm | USA | Redwood National Park | 383188 | 369726 | 383253 | 388352 | 404789 | 408126 | 392029 | 394144 | 383780 | 385171 | 389256 | https://irma.nps.gov/Stats/ (accessed 17/11/2014) |
| NAm | USA | Rocky Mountain National Park | 3035422 | 3186323 | 3185392 | 3139685 | 2988475 | 3067256 | 2781899 | 2798368 | 2743676 | 2895383 | 2982188 | https://irma.nps.gov/Stats/ (accessed 17/11/2014) |
| NAm | USA | Ross Lake NRA |  |  |  |  |  | 346542 | 313497 | 279581 | 265022 | 290701 | 299069 | https://irma.nps.gov/Stats/ (accessed 17/11/2014) |
| NAm | USA | Saguaro National Park | 716160 | 749014 | 765195 | 725874 | 615045 | 643697 | 651464 | 727208 | 619983 | 658477 | 687212 | https://irma.nps.gov/Stats/ (accessed 17/11/2014) |
| NAm | USA | Saint Croix NSR |  |  |  |  |  | 282838 | 313904 | 521695 | 506841 | 519330 | 428922 | https://irma.nps.gov/Stats/ (accessed 17/11/2014) |
| NAm | USA | Santa Monica Mountains NRA |  |  |  |  |  | 447776 | 525859 | 447190 | 534378 | 577686 | 506578 | https://irma.nps.gov/Stats/ (accessed 17/11/2014) |
| NAm | USA | Shenandoah National Park | 1473100 | 1339286 | 1419579 | 1498561 | 1389244 | 1163950 | 1261000 | 1094912 | 1076150 | 1107227 | 1282301 | https://irma.nps.gov/Stats/ (accessed 17/11/2014) |
| NAm | USA | Sleeping Bear Dunes NL |  |  |  |  |  | 1153962 | 1114615 | 1222313 | 1213026 | 1134314 | 1167646 | https://irma.nps.gov/Stats/ (accessed 17/11/2014) |
| NAm | USA | Sunset Crater Volcano NM |  |  |  |  |  | 159073 | 126911 | 229913 | 221406 | 231855 | 193832 | https://irma.nps.gov/Stats/ (accessed 17/11/2014) |
| NAm | USA | Theodore Roosevelt National Park | 448286 | 431311 | 431813 | 446609 | 471551 | 490295 | 474589 | 493198 | 435359 | 456588 | 457960 | https://irma.nps.gov/Stats/ (accessed 17/11/2014) |
| NAm | USA | Timpanogos Cave NM |  |  |  |  |  | 106030 | 107170 | 104517 | 110840 | 114737 | 108659 | https://irma.nps.gov/Stats/ (accessed 17/11/2014) |
| NAm | USA | Voyageurs National Park | 231958 | 228898 | 227371 | 243374 | 238227 | 234231 | 249041 | 234577 | 231875 | 220650 | 234020 | https://irma.nps.gov/Stats/ (accessed 17/11/2014) |
| NAm | USA | Walnut Canyon NM |  |  |  |  |  | 111465 | 126931 | 128275 | 120820 | 126187 | 122736 | https://irma.nps.gov/Stats/ (accessed 17/11/2014) |
| NAm | USA | White Sands NM |  |  |  |  |  | 492008 | 448185 | 450447 | 440927 | 437042 | 453722 | https://irma.nps.gov/Stats/ (accessed 17/11/2014) |
| NAm | USA | Wind Cave National Park | 849974 | 761717 | 668507 | 650357 | 810298 | 874167 | 593877 | 612478 | 591049 | 603158 | 701558 | https://irma.nps.gov/Stats/ (accessed 17/11/2014) |
| NAm | USA | Wrangell St Elias National Park | 27859 | 29252 | 28331 | 28643 | 40352 | 43311 | 57221 | 56224 |  |  | 38899 | https://irma.nps.gov/Stats/ (accessed 17/11/2014) |
| NAm | USA | Yellowstone National Park | 3120830 | 3131381 | 2838233 | 2758526 | 2973677 | 3019375 | 2868317 | 2835651 | 2870295 | 3151343 | 2956763 | https://irma.nps.gov/Stats/ (accessed 17/11/2014) |
| NAm | USA | Yosemite National Park | 3657132 | 3493607 | 3400903 | 3368731 | 3361867 | 3378664 | 3280911 | 3304144 | 3242644 | 3503428 | 3399203 | https://irma.nps.gov/Stats/ (accessed 17/11/2014) |
| NAm | USA | Yukon-Charley Rivers NPRES |  |  |  |  |  | 5552 | 4260 | 12784 | 12083 | 11567 | 9249 | https://irma.nps.gov/Stats/ (accessed 17/11/2014) |
| NAm | USA | Zion National Park | 2370048 | 2449664 | 2432348 | 2217779 | 2592545 | 2458792 | 2677342 | 2586665 | 2567350 | 2657281 | 2500981 | https://irma.nps.gov/Stats/ (accessed 17/11/2014) |

* Data for 21 Malagasy PAs and 4 Sri Lankan PAs were provided in confidence and so are not given here.

Region codes: Af = Africa; As/Au = Asia/Australasia; Eu = Europe; LAm = Latin America; NAm = North America.
