# Supplementary material for: Walk on the Wild Side: Estimating the Global Magnitude of Visits to Protected Areas
Source: PLoS Biol. 2015 Feb 24;13(2):e1002074. doi: 10.1371/journal.pbio.1002074 (PMC4339837; doi:10.1371/journal.pbio.1002074)
Supplement: S2 Table — (DOCX) [file pbio.1002074.s004.docx]

|  | Africa | Asia/Australasia | Europe | Latin America | North America |
| --- | --- | --- | --- | --- | --- |
| PA size (ha) | 0.079 | -0.087 | **0.507***** | -0.070 | -0.034 |
| Local population size | -0.002 | -0.182 | **0.370***** | 0.093 | -0.021 |
| PA remoteness (mins) | **-0.312**** | -0.111 | -0.107 | **-0.387***** | **-0.474***** |
| Natural attractiveness | 0.021 | **-0.274**** | 0.095 | **0.343**** | **0.281**** |
| National wealth  (2006 US$, PPP-adjusted) | **0.414***** | **0.295**** | 0.034 | 0.059 | **0.217*** |
| N | 94 | 96 | 101 | 132 | 123 |
|  |  |  |  |  |  |

Values for mean visit rate, PA size, local population size, remoteness and national wealth were all log_10_-transformed (after adding 1 to all values of mean visit rate, local population size and remoteness). The analyses for Europe exclude 10 UK National Parks (which were evident outliers).
