# Supplementary material for: Walk on the Wild Side: Estimating the Global Magnitude of Visits to Protected Areas
Source: PLoS Biol. 2015 Feb 24;13(2):e1002074. doi: 10.1371/journal.pbio.1002074 (PMC4339837; doi:10.1371/journal.pbio.1002074)
Supplement: S3 Table — (DOCX) [file pbio.1002074.s005.docx]

|  | Africa | Asia/  Australasia | Europe | | Latin America | North America |
| --- | --- | --- | --- | --- | --- | --- |
| **(A)** |  |  | rest of Europe | UK National Parks |  |  |
| Intercept | -1.865 | 3.786 | -1.600 | 0.021 | 3.427 | 3.624 |
| PA size (ha) | 0.160 | -0.099 | 0.177 | **1.538*** | -0.115 | 0.151 |
| Local population size | 0.096 | -0.268 | **0.357**** | 0.198 | -0.059 | 0.086 |
| PA remoteness (mins) | **-0.723**** | -0.166 | **-0.104***** | -0.259 | **-1.008***** | **-0.808***** |
| Natural attractiveness | 0.127 | **-0.314*** | 0.040 | -0.986 | **0.772***** | **0.345**** |
| National wealth  (2006 US$, PPP-adjusted) | **1.001***** | **0.783***** | **0.533***** | - | 0.113 | 0.163 |
| N | 94 | 96 | 111  0.538  <0.001 | | 132 | 123 |
| adjusted r^2^ | 0.222 | 0.164 |  |  | 0.229 | 0.283 |
| overall p | <0.001 | <0.001 |  |  | <0.001 | <0.001 |
|  |  |  |  |  |  |  |
| **(B)** |  |  |  |  |  |  |
| Number of PAs to which model fitted | 6592 | 12,223 | 55,448 | | 3600 | 16,375 |
| Median estimated visit rate/PA (visits/y) | 2881 | 44,932 | 20,811 | | 4016 | 352,854 |
| Total visit rate  [and 95% CIs]  (million visits/y) | 69  [28-322] | 1039  [402–3930] | 3849  [1307–12,294] | | 148  [50–788] | 3303  [1848–6537] |

(A) Terms and summary statistics for Generalised Linear Models (GLMs) predicting observed visit rates to PAs in each region. Values for mean visit rate, PA size, local population size, remoteness and national wealth were all log_10_-transformed (after adding 1 to all values of mean visit rate, local population size and remoteness). Statistically significant coefficients are shown in bold, with * = P<0.05, ** = P<0.01, *** = P<0.001. (B) Estimated visit rates for each region derived by applying its GLM to all its terrestrial PAs (excluding those <10 ha, and marine and Category I PAs).
