# Supplementary material for: Walk on the Wild Side: Estimating the Global Magnitude of Visits to Protected Areas
Source: PLoS Biol. 2015 Feb 24;13(2):e1002074. doi: 10.1371/journal.pbio.1002074 (PMC4339837; doi:10.1371/journal.pbio.1002074)
Supplement: S4 Table — (DOCX) [file pbio.1002074.s006.docx]

| **(A) Direct expenditure** | | | | | | | |
| --- | --- | --- | --- | --- | --- | --- | --- |
| **Region** | **Country** | **Site(s)** | **Value (2014 US$)** | **Per visit or per visit-day?** | **Method** | **Comments** | **Source** |
| Af | BWA | Okavango Delta | 481 | visit |  |  | [71] |
| Af | BWA | All PAs | 2518 | visit |  |  | [72] |
| Af | NAM | All NPs | 367 | visit-day |  |  | [1] |
| Af | NAM | All PAs + surrounding areas | 915 | visit |  |  | [73] |
| Af | RWA | PN des Volcans | 2562 | visit |  |  | [74] |
| Af | ZAF | Kruger NP | 134 | visit |  | nationals only | [75] |
| Af | ZAF | Kruger NP | 2247 | visit |  | foreigners only | [75] |
| Af | TZA | Tanzanian northern circuit | 378 | day |  |  | [76] |
| Af | UGA | Bwindi Impenetrable NP | 321 | visit |  |  | [77] |
| Af | UGA | Mgahinga Gorilla NP and Bwindi Impenetrable NP | 1131 | visit |  |  | [78] |
| As/Au | AUS | All NPs and SPs* | 141 | visit-day |  | nationals only | [79] in [80] |
| As/Au | AUS | All NPs and SPs* | 89 | visit-day |  | foreigners only | [79] in [80] |
| As/Au | AUS | Gascoyne coast region | 70 | visit-day |  |  | [81] |
| As/Au | AUS | Grampians NP | 54 | visit-day |  |  | [82] in [80] |
| As/Au | AUS | Part of New South Wales public lands | 93 | visit-day |  | nationals only | [83] |
| As/Au | AUS | Part of New South Wales public lands | 81 | visit-day |  | foreigners only | [83] |
| As/Au | AUS | Southern forests, Western Australia | 78 | visit-day |  |  | [81] |
| As/Au | AUS | Watarraka NP | 146 | visit |  |  | [84] in [80] |
| As/Au | IDN | Komodo NP | 284 | visit |  |  | [85] |
| As/Au | NPL | All PAs | 507 | visit |  |  | [86] |
| As/Au | NPL | Annapurna NP | 395 | visit |  |  | [87] |
| As/Au | NPL | Phulchoki Important Bird Area | 8 | visit |  |  | [88] |
| As/Au | NPL | Shivapuri-Nagarjun NP | 26 | visit |  |  | Merriman, J., pers. comm. |
| As/Au | PAK | Margalla Hills NP | 40 | visit |  |  | [89] |
| Eu | ITA | Griffon vulture project area | 9 | visit |  |  | [90] |
| Eu | ITA | NP of the Dolomiti Bellenusi | 17 | visit |  |  | [90] |
| Eu | ITA | NP of the Dolomiti Ampezzane | 47 | visit |  |  | [90] |
| Eu | ITA | Quadris Nature Area | 3 | visit |  |  | [90] |
| Eu | SWE | Arjeplog forest | 889 | visit |  |  | [91] |
| Eu | SWE | Harasjömåla forest | 470 | visit |  |  | [91] |
| Eu | GBR | All visits to English countryside * | 29 | visit |  |  | [57] |
| Eu | GBR | Wicken Fen (479 ha restoration site) | 13 | visit |  |  | Peh, K., pers. comm. |
| LAm | BLZ | Cockscombe Basin Wildlife Sanctuary | 3 | visit |  |  | [92] |
| LAm | BLZ | Possum Point | 745 | visit |  |  | [93] |
| LAm | CRI | Monteverde Cloud Forest Reserve | 311 | visit |  |  | [94] |
| LAm | CRI | Ostional Wildlife Refuge | 12 | visit |  |  | [95] |
| LAm | ECU | Cuyabeno Wildlife Reserve | 46 | visit |  |  | [96] |
| LAm | ECU | Galapagos NP | 701 | visit |  |  | [97] |
| LAm | ECU | Galapagos NP | 2287 | visit |  |  | [98] |
| LAm | MSR | Centre Hills Reserve | 187 | visit |  | foreigners only | Peh, K., pers. comm. |
| LAm | PER | Madre de Dios | 462 | visit |  |  | [99] |
| LAm | PER | Manu NP | 42 | visit-day |  |  | [100] |
| LAm | PER | Tambopata National Reserve | 355 | visit |  |  | [101] |
| NAm | CAN | Algonquin Provincial Park | 74 | visit-day |  |  | [102] in [2]; [103] |
| NAm | CAN | Bruce Peninsula NP | 103 | visit-day |  |  | [104] in [4] |
| NAm | CAN | Commercial nature-based tourism, British Columbia | 325 | visit-day |  |  | [105] |
| NAm | CAN | Pukaskwa NP | 163 | visit-day |  |  | [104] in [4] |
| NAm | CAN | Remote fishing sites, northern Ontario | 896 | visit |  |  | [106] |
| NAm | USA | All NPs* | 46 | visit |  |  | [107] |
| *NAm* | USA | Mt Rainier NP | 31 | visit |  |  | [108] |
| *medians (N)* | **Africa (10)** | | **698** |  |  |  |  |
|  | **Asia/Australasia (14)** | | **85** |  |  |  |  |
|  | **Europe (8)** | | **23** |  |  |  |  |
|  | **Latin America (11)** | | **311** |  |  |  |  |
|  | **North America (7)** | | **103** |  |  |  |  |
|  | | | | | | | |
| **(B) Consumer surplus** | | | | | | | |
| **Region** | **Country** | **Site(s)** | **Value (2014 US$)** | **Per visit or per visit-day?** | **Method** | **Comments** | **Source** |
| Af | BWA | All PAs | 517 | visit | CV |  | [72] |
| Af | KEN | All NPs and Game Reserves | 121 | visit | CV |  | [109] |
| Af | KEN | All PAs | 889 | visit | TC | foreigners only | [110] |
| Af | KEN | All PAs | 721 | visit | CV | foreigners only | [110] |
| Af | KEN | Lake Nakuru NP | 92 | visit | CV |  | [111] |
| Af | KEN | Lake Nakuru NP | 133 | visit | TC | nationals only | [111] |
| Af | KEN | Lake Nakuru NP | 203 | visit | TC | foreigners only | [111] |
| Af | MDG | Beza Mahafaly Special Reserve | 575 | visit | TC | foreigners only | [112] |
| Af | MDG | Mantadia NP | 43 | visit | TC | foreigners only | [113] |
| Af | MDG | Mantadia NP | 118 | visit | CV | foreigners only | [113] |
| Af | MDG | Mantadia NP | 111 | visit | CV |  | [114] |
| Af | NAM | All PAs + surrounding areas | 153 | visit | CV | nationals only | [73] |
| Af | NAM | All PAs + surrounding areas | 262 | visit | CV | foreigners only | [73] |
| Af | TZA | Tarangire NP | 27 | visit | CV | non-residents only | [115] in [116] |
| Af | TZA | Tarangire NP | 10 | visit | CV | foreign residents only | [115] in [116] |
| Af | UGA | Mgahinga Gorilla NP and Bwindi Impenetrable NP | 269 | visit | TC + CV |  | [78] |
| Af | ZWE | Hwange NP and Mana Pools NP | 663 | visit | TC + CV | foreigners only | [109] in [108] |
| As/Au | IDN | Komodo NP | 17 | visit | CV |  | [118] |
| As/Au | IRN | Lahijan Forest | 1 | visit | CV |  | [119] |
| As/Au | NPL | Annapurna NP | 49 | visit | CV |  | [87] |
| As/Au | PAK | Margalla Hills NP | 5 | visit | TC |  | [89] |
| As/Au | KOR | Mt Kayasan NP | 17 | visit | CV |  | [120] |
| As/Au | KOR | Mt Minju | 11 | visit | CV |  | [121] |
| As/Au | KOR | Mt Pukansan NP | 13 | visit | CV |  | [120] |
| As/Au | KOR | Seoraksan NP | 20 | visit | CV |  | [120] |
| As/Au | KOR | Taean-Haean NP | 7 | visit | CV |  | [120] |
| As/Au | THA | Khao Yai NP | 54 | visit | TC | nationals only | [122] |
| Eu | CZE | Bile Karpaty Landscape PA | 9 | visit | TC + CV |  | [123] |
| Eu | ITA | Campo Grosso | 5 | visit | CV |  | [124] |
| Eu | ITA | Griffon vulture project area | 6 | visit | TC + CV |  | [90] |
| Eu | ITA | NP of the Dolomiti Bellenusi | 8 | visit | TC + CV |  | [90] |
| Eu | ITA | Natural Park of the Dolomiti Ampezzane | 7 | visit | TC + CV |  | [90] |
| Eu | ITA | Quadris Nature Area | 2 | visit | TC + CV |  | [90] |
| Eu | SWE | Arjeplog forest | 499 | visit | CV |  | [91] |
| Eu | SWE | Harasjömåla forest | 154 | visit | CV |  | [91] |
| Eu | GBR | All Great Britain ecosystems* | 5 | visit | CV |  | [15] |
| LAm | CRI | 3 NPs | 37 | visit | CV | foreigners only | [125] |
| LAm | CRI | Monteverde Cloud Forest Reserve | 70 | visit | TC | nationals only | [126] |
| LAm | CRI | Monteverde Cloud Forest Reserve | 2079 | visit | TC | US visitors only | [127] |
| LAm | CRI | Monteverde Cloud Forest Reserve | 210 | visit | CV |  | [128] |
| NAm | USA | 20 studies of wilderness areas* | 60 | visit-day | TC + CV |  | [129] |
| NAm | USA | 37 studies of outdoor recreation* | 51 | visit-day | TC + CV |  | [130] |
| NAm | USA | Apalachicola River region, Florida | 102 | visit-day | TC |  | [131] |
| NAm | USA & CAN | All wildlife viewing* | 53 | visit-day | TC + CV |  | <http://recvaluation.forestry.oregonstate.edu/sites/default/files/RECREATION_USE_VALUES_DATABASE_%20SUMMARY.pdf> |
| *medians (N)* | **Africa (17)** | | **153** |  |  |  |  |
|  | **Asia/Australasia (10)** | | **15** |  |  |  |  |
|  | **Europe (9)** | | **7** |  |  |  |  |
|  | **Latin America (4)** | | **140** |  |  |  |  |
|  | **North America (4)** | | **56** |  |  |  |  |

(A) Estimates of direct expenditure are within-country and exclude indirect or induced expenditure. (B) Estimates of consumer surplus are based on travel cost (TC) or contingent valuation (CV) methods, or both. Region-specific medians are given at the bottom of each section; note that these are reasonably close to values reported by studies from the same region based on very large samples or meta-analyses (marked with asterisks). Countries are given using their ISO 3166-1 alpha-3 code. PA = protected area; NP = National Park; SP = State Park.
