# Supplementary material for: Walk on the Wild Side: Estimating the Global Magnitude of Visits to Protected Areas
Source: PLoS Biol. 2015 Feb 24;13(2):e1002074. doi: 10.1371/journal.pbio.1002074 (PMC4339837; doi:10.1371/journal.pbio.1002074)
Supplement: S1 Text — (DOCX) [file pbio.1002074.s007.docx]

**Supporting Information**

**Materials and Methods**

**Obtaining Data on Visit Rates**

We interpreted nature-based recreation and tourism (after [1]) as visiting a relatively undisturbed natural area in order to enjoy, admire or study its fauna, flora or scenery, either exclusively or in conjunction with activities such as walking, canoeing or mountain-biking. We measured its extent by compiling counts of visits to terrestrial Protected Areas (PAs), drawn from the peer-reviewed and grey literature, online datasets and personal contacts (S1 Table).

Such data are scattered and noisy. The methods they derive from vary widely and include dedicated studies, gate receipts, and automated trail and road counts [2, 3]. However most records we obtained were not accompanied by details of how they were collected and so we were unable to adjust for this variation in any systematic way, and instead chose to deal with the inherent noise by using all available data (with some important exceptions – see below). Where possible we scored each visitor-day (when a person spends at least a portion of a day at a site) as a visit (following [4, 5]), although in an unknown proportion of PAs longer and multiple-entry stays are counted as single visits. There may be some biases in visit data, with corruption, for example, probably leading to systematic under- (and in some cases over-) reporting of visits [6]. However we think it is unlikely such errors are in a consistent direction, overall or among regions.

To avoid being confounded by pervasive temporal trends in visitor numbers [7, 8] we restricted analyses to our best sampled decade, from 1998 to 2007. We excluded any PAs not included in the World Database on Protected Areas (WDPA) [9], as well as marine PAs (identified as such in the WDPA, or with >50% of their area falling in the sea) and those in Antarctica (as they host very different forms of recreation). Because PAs in IUCN Categories Ia and Ib are strictly protected and typically do not encourage visitors [10] we excluded Category I sites from our dataset. Where evident from their names we excluded largely cultural PAs (e.g. the USA’s National Historic Sites, National Memorials and National Battlefields).

Our resulting sample comprised 2663 records of annual visits to 556 PAs from 51 countries (S1 Table). The overall geographical spread of data was reasonable (Fig. 1), but noticeably patchy for island states, and across much of the Middle East and North Africa (a region where there is generally limited biodiversity information and marked under-investment in conservation [11, 12, 13]). Averaged across the years for which we had data, mean visit rates per PA varied enormously, from zero recorded visits to several PAs in Africa and remote parts of Chile, to three PAs (Golden Gate National Recreation Area, USA; and the UK’s Lake District and Peak District National Parks) with over 10 million visits/y.

**Potential Predictors of Visit Rates**

For each PA in our sample we derived five variables which we considered might plausibly predict variation in mean annual visit rates. All mapping work to extract these was performed in ArcGIS version 9.3.1 [14]. The locations of PAs were taken from the World Database on Protected Areas (WDPA) [9]. Many PAs are still presented as points in the WDPA so to obtain potential predictor variable values for each of these we created a circular polygon of the same area as the PA, centred on the point given.

***Protected area size:*** we expected larger PAs to have higher visit rates. We extracted the area of each PA (in ha) from the WDPA [9].

***Local population size:*** we expected PAs with more people living near them would experience higher visit rates [15, 16, 17]. We estimated the total number of people within 100km of each PA’s perimeter using the ‘Zonal Stats’ tool in ArcMap and the Gridded Population of the World [18]. Zones were defined as 100km buffers around WDPA shapefiles (or around our circular polygons, for PAs presented only as points).

***Protected area remoteness:*** we expected visit rates to decrease with increasing remoteness of PAs [15-17, 19]. We took as our measure of remoteness the shortest time taken (in minutes) to travel to the nearest city with a population of >50,000 in 2000, using a global friction surface [20].

***Natural attractiveness****:* we expected PAs with more attractive natural features to have higher visit rates [16, 19, 21, 22], though note that such associations are often quite weak [15]. To derive a simple measure of natural attractiveness which could be easily estimated for each PA, three experienced conservation scientists (AB, AM, and Neil Burgess) independently scored (on a 1–5 scale) the likely attractiveness for nature-focused visitors of each of up to 14 biomes in each of eight terrestrial realms [23]. This score was based on the mammals and birds that might plausibly be seen by a visitor. There was reasonable agreement across experts in these scores (Spearman rank correlations on scores for N=65 biome-realm combinations: r_s_=0.85, 0.62, 0.51; all P< 0.001). Each PA was then assigned the mean score for the biome-realm combination it occurred in (assessed by intersecting the WDPA, MHT and realm shapefiles). PAs which overlapped more than one biome received their mean score, but with +1 added to reflect the diversity of habitat types present. We explored more detailed approaches to quantifying the attractiveness of the natural features of PAs, such as the number of species or threatened species at a site, or the presence of particular charismatic species or geological features, but the limited availability of such data for individual PAs made this impossible for most of our PA sample.

***National income:*** we expected PAs in richer countries to have higher visit rates [16]. As a measure of the size of the economy within which our sample PAs were located we took each country’s GDP for 2006, adjusted for Purchasing Power Parity and expressed in 2006 US$, from the Euromonitor website [24].

Other candidate predictor variables we initially considered (the quality of road infrastructure; distance from a major airport; the prevalence of malaria; and the incidence of armed conflict) co-varied with GDP or varied little within regions and so were dropped. Except for Category I PAs generally excluding visitors, IUCN categories are not closely linked to how far PAs are focused on tourism and so were considered unlikely to be informative.

**Understanding Variation in Visit Rates**

To achieve approximate normality, prior to statistical analysis values for mean visit rate, PA size, local population size, remoteness and national income were all log_10_-transformed (after adding 1 to all values of mean visit rate, local population size and remoteness). Transformed visit rates were then regressed against transformed values of each potential predictor variable (S2 Table). Initial inspection of the data suggested substantial geographical variation in relationships so we performed these univariate regressions separately for five regions: Africa (N=94), Asia/Australasia (including Russia) (96), Europe (101), Latin America (including the Caribbean) (132) and North America (123); the Europe regressions excluded data for exceptionally highly-visited UK National Parks (10, including The Broads Authority) as these were evident outliers.

In order to better understand the independent associations between our visit rates and our potential predictor variables we next built a series of General Linear Models, one for each region. For Europe, we allowed the fitted coefficients for the UK National Parks to be different for this very unusual subset of PAs by adding the appropriate interaction terms. In order to maximise our explanatory and predictive power, all variables were kept the in the model – i.e. we performed no model simplification, as this might have led to a loss of information when trying to predict PA visits across the world. Because of the number of variables involved relative to our sample sizes, we did not explore interaction terms among predictors.

**Estimating PA Visit Rates Worldwide**

We applied our region-specific GLMs to the PAs listed in the WDPA [9], again excluding all marine, Antarctic and Category I sites. This is the only global catalogue of PAs, but is acknowledged to have many gaps [25] – listing, for example, more than 10,000 PAs for the UK but only 724 for the whole of China (cf 1865 National Parks summarised in [26]). Such errors of omission could not be addressed but mean that our aggregate estimates of visit rates are probably conservative.

For each remaining PA in the dataset we extracted values for each of our predictor variables (as above). Within each region the range of values across these PAs was well represented by the values of the sample PAs used to build the GLMs (S2 Fig.), with one exception. We had no visit rate data for sites <10 ha in size, even though 39,476 of the WDPA sites are smaller than this; with no empirical basis for estimating their visit rates, we dropped these extremely small sites (which together account for <0.01% of global PA extent [9]) from further analysis. As a further precaution to avoid assuming that fitted relationships with predictor variables held beyond the range covered by our sample sites, in applying our GLMs we constrained those PAs with higher (or lower) values for those variables to take the maximum (or minimum) values of the sample sites in their region.

We used our regional GLM results to predict annual visit rates for each of 94,238 PAs (6592 in Africa, 12,223 in Asia/Australasia, 55,448 in Europe, 3600 in Latin America and 16,375 in North America). We then estimated median and total visit rates across all PAs in each country, and summed the national totals to give estimated total visit rates for each region and for the world. To generate 95% confidence intervals (CIs) of our estimates, we bootstrapped our visit information for each continent 1000 times, re-fitted our GLMs to the bootstrapped datasets and then re-predicted visits for the whole WDPA, taking the 2.5% and 97.5% quantiles of the estimates as our CIs. Inspection of diagnostic plots revealed no points have leverage >1 in any model, so we considered bootstrapping to be more appropriate than jackknifing for exploring model uncertainty.

**Estimating Numbers of Non-work Person-days**

We estimated each country’s number of non-work person-days available for recreation in PAs as the sum of the number of days of paid annual leave (from [27]) and of weekend days (=104/y), multiplied by the country’s total population (in 2003, the mid-point of our analysis, from [28]). We then summed these figures by region and compared them with estimates of region-wide PA visit rates to derive region-specific estimates of PA visit rates/100 non-work person-days. These estimates are only indicative: because of insufficient data we could not reallocate non-domestic visits to visitors’ country of origin; and the number of non-work days for those in paid employment will be an imperfect measure of average recreation opportunities in many developing countries, for children, and for those above working age.

**Valuing Visits**

We searched the peer-reviewed and grey literature and online databases for empirically-derived estimates of the economic values of visits to terrestrial protected areas or similar natural sites (S4 Table). We extracted two different sorts of measures – those of direct expenditure (as a measure of economic impact; N=50 estimates), and of consumer surplus (as a measure of economic value of the trip to the visitor; N=44 estimates). To be conservative, where possible, direct expenditure estimates were of spend within a country (so excluded international travel, for example), and excluded indirect expenditure (e.g. government and private sector investment) and induced expenditure (spending by those employed in the sector) [29]. Few studies reported net expenditure, so for consistency the figures here are all gross. Consumer surplus estimates were based on travel cost or contingent valuation (= willingness-to-pay) methods, or both. Estimates were expressed per visit-day wherever possible (to be conservative) and otherwise per visit. All values were converted to US$ at the time of the study [30], and then to 2014 US$ [31].

Available data on both direct expenditure and consumer surplus were too sparse and confounded by variation in methods to permit formal statistical analysis of variation in values and so instead we adopted a simpler approach, making use of all available information. We again looked at regional variation, and found for both measures that median reported values were lowest in Europe, more in Asia/Australasia and North America, higher in Latin America, and highest of all in Africa (see S4 Table for regional medians). This may be related to regional differences in sites (European PAs tend to more accessible and smaller than others, while Latin American and African PAs are typically bigger and more attractive – S2 Fig.) and in the origins of their visitors (when data are split, foreign visitors tend to have more valuable visits [S4 Table], and data collated in compiling S1 Table showed the % of PA visitors who were foreign was least in Asia/Australasia [13%, N = 82 records], higher in Latin America [20%, N = 92], and greatest in Africa [61%, N = 57]). However we lack sufficient information on visitor profiles from the valuation studies to test these ideas formally.

Given these apparent regional patterns we estimated global direct expenditure and consumer surplus associated with PA visits by multiplying observed median values per visit for each region by their estimated total visit rates, and then summing these totals across regions.

**References**

1. Turpie J, Barnes J, Lange G-M, Martin P (2010) The Economic Value of Namibia’s Protected Area System. Windhoek: Ministry of Environment and Tourism. 58 p.
2. Eagles PFJ (2003) International trends in park tourism. George Wright Forum 20: 25-57.
3. Cessford G, Burns R (2008) Monitoring Visitor Numbers in New Zealand National Parks and Protected Areas. Wellington: Department of Conservation. 46 p.
4. Eagles PFJ, McLean D, Stabler MJ (2000) Estimating the tourism volume and value in protected areas in Canada and the USA. George Wright Forum 17: 62-76.
5. Wood SA, Guerry AD, Silver JM, Lacayo M (2013) Using social media to quantify nature-based tourism and recreation. Sci Rep 3: 2976.
6. Cochrane J (2003) Ecotourism, Conservation and Sustainability: A Case Study of Bromu Tengger Semeru National Park, Indonesia. Ph.D. Thesis, University of Hull.
7. Pergams ORW, Zaradic PA (2008) Evidence for a fundamental and pervasive shift away from nature-based recreation. Proc Natl Acad Sci USA 105: 2295-2300.
8. Balmford A, et al. (2009) A global perspective on trends in nature-based tourism. PLoS Biol 7: e1000144.
9. IUCN, UNEP (2012) The World Database on Protected Areas. Available at: http://www.[protectedplanet.net](http://protectedplanet.net).
10. Dudley N (2013) Guidelines for Applying Protected Area Management Categories*.* Gland: IUCN. 86 p.
11. Waldron A, et al. (2013) Targeting global conservation funding to limit immediate biodiversity declines. Proc Natl Acad Sci USA 110: 12144-12148.
12. Amano T, Sutherland WJ (2013) Four barriers to the global understanding on biodiversity conservation: wealth, language, geographical location and security. Proc R Soc B 280: 20122649.
13. Durant SM, et al. (2014) Fiddling in biodiversity hotspots while deserts burn? Collapse of the Sahara’s megafauna. Divers Distrib 20: 114-122.
14. ESRI (2009) ArcGIS Desktop: Release 9.3.1 with Spatial Analyst Extension. California: Redlands.
15. Sen A, et al. (2012) Economic Assessments of the Recreational Value of Ecosystems in Great Britain. Norwich: CSERGE, University of East Anglia. 63 p.
16. Ghermandi A, Nunes PALD (2013) A global map of recreation values: results from a spatially explicit meta-analysis. Ecol Econ 86: 1-15.
17. Brainard J, Bateman I, Lovett A (2001) Modelling demand for recreation in English woodlands. Forestry 74: 423-438.

Center for International Earth Science Information Network, United Nations Food and Agriculture Programme, Centro Internacional de Agricultura Tropical (2005) Gridded population of the world: Future estimates. Available at: <http://sedac.ciesin.columbia.edu/gpw>.

1. Neuvonen M, Pouta E, Puustinen J, Sievänen T (2010) Visits to national parks: effects of park characteristics and spatial demand. J Nat Conserv 18: 224-229.

Nelson A (2008) Estimated travel time to the nearest city of 50,000 or more people in year 2000: Available at: http://bioval.jrc.ec.europa.eu/products/gam/download.htm.

1. Williams ID, Polunin NVC (2000) Differences between protected and unprotected reefs of the western Caribbean in attributes preferred by dive tourists. Environ Conserv 27: 382-391.
2. Naidoo R, Adamowicz WL (2005) Biodiversity and nature-based tourism at forest reserves in Uganda. Environ Dev Econ 10: 159-178.
3. Olson DM, et al. (2001) Terrestrial ecoregions of the world: a new map of life on Earth. Bioscience 51: 933-938.
4. Euromonitor International (2011) Passport. Available at: <http://www.euromonitor>.com/.
5. Visconti P, et al. (2013) Effects of errors and gaps in spatial data sets on assessment of conservation progress. Conserv Biol 27: 1000-1010.
6. Wang G, et al. (2012) National park development in China: conservation or commercialization? Ambio 41: 247-261.
7. International Labour Organization (2013) Working Conditions Laws Report 2012. Geneva: ILO. 90 p.
8. United Nations (2004) World Urbanization Prospects: The 2003 Revision. New York: United Nations.
9. World Travel & Tourism Council (2012) Travel and Tourism: Economic Impact. London: WTTC. 20 p.
10. OANDA (2014) Currency converter. Available at: http://www.oanda.com/currency/converter/.
11. United States Department of Labor (2014) CPI inflation calculator. Available at: <http://data.bls.gov/cgi-bin/cpicalc.pl>.
12. Mbaiwa JE, Darkoh MBK (2008) The socio-economic and environmental effects of the implementation of the Tourism Policy of 1990 in the Okavango Delta, Botswana. Botswana Notes & Records 39: 138-155.
13. Thorsell J, Sigaty T (1998) Human Use of World Heritage Natural Sites – a Global Overview. Gland: IUCN. 47 p.
14. Kuuder, CJW, et al. (2013) Assessment of visitor satisfaction in Mole National Park, Ghana. African Journal of Hospitality, Tourism and Leisure 2(3). ISSN: 2223-814X.
15. Rwanda Development Board (2011) Highlights on National Parks Visitation in Rwanda: January – March 2011. Kigali: Rwanda Development Board. 8 p.
16. Tanzania National Parks (2002) TANAPA Quick Reference Statistics. Dar es Salaam: Tanzania National Parks.
17. Baldus RD, et al. (2003) Seeking conservation partnerships in the Selous Game Reserve, Tanzania. PARKS 13(1): 50-61.
18. National Environment Management Authority (2004) State of the Environment Report for Uganda 2004/05. Kampala: National Environmental Management Authority. 301 p.
19. Uganda Wildlife Authority (2006) Operation of Launches/Boats, Murchison Falls National Park. Kampala: Uganda Wildlife Authority.
20. Buckley R (2002) World Heritage Icon Value: Contribution of World Heritage Branding to Nature Tourism. Canberra: Australian Heritage Commission.
21. UNEP-WCMC (2011) Huanglong Scenic and Historic Interest Area: China. World Heritage Information Sheet. Cambridge: UNEP-WCMC. Available at: http://www.unep-wcmc.org/resources-and-data/world-heritage-information-sheets
22. UNEP-WCMC (2011) Mount Huangshan: China. World Heritage Information Sheet. Cambridge: UNEP-WCMC. Available at: http://www.unep-wcmc.org/resources-and-data/world-heritage-information-sheets
23. UNEP-WCMC (2011) Sichuan Giant Panda Sanctuaries – Wolong, Mt. Siguniang and Jiajin Mountains: China. World Heritage Information Sheet. Cambridge: UNEP-WCMC. Available at: http://www.unep-wcmc.org/resources-and-data/world-heritage-information-sheets
24. UNEP-WCMC (2011) Three Parallel Rivers of Yunnan Protected Areas: China. World Heritage Information Sheet. Cambridge: UNEP-WCMC. Available at: http://www.unep-wcmc.org/resources-and-data/world-heritage-information-sheets
25. Liu W, et al. (2012) Drivers and socioeconomic impacts of tourism participation in protected areas. PLoS ONE 7(4): e35420.
26. Karanth KK, DeFries R (2011) Nature-based tourism in Indian protected areas: new challenges for park management. Conserv Lett 4: 137-149.
27. Project Tiger (2005) Joining the Dots. New Delhi: Union Ministry of Environment and Forests. 206 p.
28. Martin EB (2006) Policies that work for rhino conservation in West Bengal. Pachyderm 41: 74-84.
29. Naicker K (2005) Balancing the Books Between the 3 Es (Environment, Ecotourism and Economics). M.Sc. Thesis, University of Kent.
30. Cochrane J 2003 (2003) Ecotourism, Conservation and Sustainability: A Case Study of Bromo Tengger Semeru National Park, Indonesia. Ph.D. Thesis, University of Hull.
31. Ministry of Natural Resources and Environment (2006) Management Effectiveness Assessment of National and State Parks in Malaysia. Putrajaya: Ministry of Natural Resources and Environment. 32 p.
32. UNEP-WCMC (2011) Kinabalu Park: Malaysia. World Heritage Information Sheet. Cambridge: UNEP-WCMC. Available at: http://www.unep-wcmc.org/resources-and-data/world-heritage-information-sheets
33. Department of Conservation (2005) Nelson/Marlborough Visitor Statistics 1 July 2003 – 30 June 2004. Factsheet 153. Nelson: Department of Conservation. 7 p.
34. UNEP-WCMC (2011) Dong Phayayen-Khao Yai Forest Complex: Thailand. World Heritage Information Sheet. Cambridge: UNEP-WCMC. Available at: http://www.unep-wcmc.org/resources-and-data/world-heritage-information-sheets
35. UNEP-WCMC (2012) Phong Nha-Ke Bang National Park: Vietnam. World Heritage Information Sheet. UNEP-WCMC: Cambridge UK. Available at: http://www.unep-wcmc.org/resources-and-data/world-heritage-information-sheets
36. UNEP-WCMC (2011) Skocjan Caves: Slovenia. World Heritage Information Sheet. UNEP-WCMC: Cambridge UK. Available at: http://www.unep-wcmc.org/resources-and-data/world-heritage-information-sheets
37. Natural England (2006) England Leisure Visits – Report of the 2005 Survey. Peterborough: Natural England. 89 p.
38. UNEP-WCMC (2011) Ischigualasto-Talampaya Natural Parks: Argentina. World Heritage Information Sheet. Cambridge: UNEP-WCMC. Available at: http://www.unep-wcmc.org/resources-and-data/world-heritage-information-sheets
39. Brown CR (2001) Visitor Use Fees in Protected Areas*.* Arlington, VA: The Nature Conservancy. 67 p.
40. Buckley R (2010) Conservation Tourism. CABI: Wallingford.
41. Drumm A (2004) Evaluation of the Pilot Fee System at Eduardo Avaroa Reserve and Recommendations for the Bolivian Protected Area System. Arlington, VA: The Nature Conservancy. 46 p.
42. UNEP-WCMC (2012) Central Amazon Conservation Complex: Brazil. World Heritage Information Sheet. Cambridge: UNEP-WCMC. Available at: <http://www.unep-wcmc.org/resources-and-data/world-heritage-information-sheets>
43. Instituto Nacional de Estadisticas (2000) Anuario de Turismo 1999. Santiago: Instituto Nacional de Estadisticas. 64 p.
44. Instituto Nacional de Estadisticas (2001) Anuario de Turismo 2000. Santiago: Instituto Nacional de Estadisticas. 80 p.
45. Instituto Nacional de Estadisticas (2002) Anuario de Turismo 2001. Santiago: Instituto Nacional de Estadisticas. 83 p.
46. Instituto Nacional de Estadisticas (2003) Anuario de Turismo 2002. Santiago: Instituto Nacional de Estadisticas. 91 p.
47. Instituto Nacional de Estadisticas (2004) Anuario de Turismo 2003. Santiago: Instituto Nacional de Estadisticas. 88 p.
48. Instituto Nacional de Estadisticas (2005) Anuario de Turismo 2004. Santiago: Instituto Nacional de Estadisticas. 97 p.
49. Instituto Nacional de Estadisticas (2006) Turismo Informe Annual 2005. Santiago: Instituto Nacional de Estadisticas. 87 p.
50. UNEP-WCMC (2011) Historic Sanctuary of Machu Picchu: Peru. World Heritage Information Sheet. Cambridge: UNEP-WCMC. Available at: http://www.unep-wcmc.org/resources-and-data/world-heritage-information-sheets
51. Mmopelwa G, Blignaut JN (2006) The Okavango Delta: the value of tourism. South African J Econ Manage Sci 9: 113-127.
52. Barnes JI (1996) Economic characteristics of demand for wildlife-viewing tourism in Botswana. Dev South Afr 13: 377-397.
53. Barnes JI, Schier C, van Rooy G (1997) Tourists’ Willingness to Pay for Wildlife Viewing and Wildlife Conservation in Namibia. Windhoek: Directorate of Environmental Affairs. 26 p.
54. Lindberg K (1991) Policies for Maximizing Nature Tourism’s Ecology and Economic Benefits. Washington, DC: World Resources Institute. 37 p.
55. Engelbrecht WG, van der Walt PT (1993) Notes on the economic use of the Kruger National Park. Koedoe 36: 113-119.
56. Sekar N, Weiss JM, Dobson AP (2014) Willingness-to-pay and the perfect safari: valuation and cultural evaluation of safari package attributes in the Serengeti and Tanzanian Northern Circuit. Ecol Econ 97: 34-41.
57. Sandbrook CG (2010) Local economic impact of different forms of nature-based tourism. Conserv Lett 3: 21-28.
58. Moyini Y, Uwimbabazi B (2000) Analysis of the Economic Significance of Gorilla Tourism in Uganda. Kampala: International Gorilla Conservation Programme. 72 p.
59. Tourism Research Australia (2008) National and State Parks Factsheet 2007. Canberra: Tourism Research Australia.
60. Driml S (2010) The Economic Value of Tourism to National Parks and Protected Areas in Australia. Gold Coast: Sustainable Tourism Cooperative Research Centre. 51 p.
61. Carlsen J, Wood D (2004) Assessment of the Economic Value of Recreation and Tourism in Western Australia’s National Parks, Marine Parks and Forests*.* Gold Coast: Sustainable Tourism Cooperative Research Centre. 29 p.
62. Hughes M, et al. (2009) Estimating the Economic, Social and Environmental Value of Tourism to Protected Areas. Gold Coast: Sustainable Tourism Cooperative Research Centre. 80 p.
63. Carlsen J (1997) Economic evaluation of recreation and tourism in natural areas: a case study in New South Wales, Australia. Tourism Economics 3: 227-239.
64. Tremblay P, Carson D (2007) Tourism and the Economic Valuation of Parks and Protected Areas: Watarrka National Park, Northern Territory. Gold Coast: Sustainable Tourism Cooperative Research Centre. 68 p.
65. Walpole MJ, Goodwin HJ (2000) Local economic impacts of dragon tourism in Indonesia. Ann Tourism Res 27: 559-576.
66. Wells MP (1993) Neglect of biological riches: the economics of nature tourism in Nepal. Biodivers Conserv 2: 445-464.
67. Baral N, Stern MJ, Bhattarai R (2008) Contingent valuation of ecotourism in Annapurna conservation area, Nepal: implications for sustainable park finance and local development. Ecol Econ 66: 218-227.
68. Birch J, et al. (2014) What benefits do community forests provide, and to whom? A rapid assessment of ecosystem services from a Himalayan forest, Nepal. Ecosyst Serv <http://dx.doi.org/10.1016/j.ecoser.2014.03.005>.
69. Khan H (2004) Demand for Eco-Tourism: Estimating Recreational Benefits from the Margalla Hills National Park in Northern Pakistan. Kathmandu: South Asian Network for Development and Environmental Economics. 38 p.
70. Tempesta T, Visintin F, Marangon F (2002) Ecotourism demand in north-east Italy. In: Arberger A, Brandenburg C, Muhar A, editors. Monitoring and Management of Visitor Flows in Recreational and Protected Areas. Vienna: Bodenkultur University. pp. 373*–*379.
71. Bostedt G, Mattson L (1995) The value of forests for tourism in Sweden. Ann Tourism Res 22: 671-680.
72. Lindberg K, Enriquez J, Sprowle K (1996) Ecotourism questioned. Case studies from Belize. Ann Tourism Res 23: 543-562.
73. Kangas P, Shave M, Shave P (1995) Economics of an ecotourism operation in Belize. Env Manage 19: 669.
74. Aylward B, Allen K, Echeverría J, Tosi J (1996) Sustainable ecotourism in Costa Rica: the Monteverde Cloud Forest. Biodivers Conserv 5: 315-343.
75. Campbell LM (1999) Ecotourism in rural developing communities. Ann Tourism Res 26: 534-553.
76. Wunder S (2000) Ecotourism and economic incentives – an empirical approach. Ecol Econ 32: 465-479.
77. Taylor JE, Hardner J, Stewart M (2006) Ecotourism and Economic Growth in the Galapagos: an Island Economy-wide Analysis. Davis: University of California. 31 p.
78. Charles Darwin Research Station (2001) Tourism and Conservation Partnerships – a View for Galapagos. Isla Santa Cruz, Galapagos Islands: Charles Darwin Research Station.
79. Groom MJ, Podolsky RD, Munn CA (1991) Tourism as a sustained use of wildlife: a case study of Madre de Dios, southeastern Peru. In: Robinson, JG, Redford KH, editors. Neotropical Wildlife Use and Conservation. Chicago: University of Chicago Press. pp. 393–412.
80. Ohl-Schacherer J, Mannigel E, Kirkby C, Shepard Jr, GH, Yu DW (2008) Indigenous ecotourism in the Amazon: a case study of ‘Casa Matsiguenka’ in Manu National Park, Peru. Environ Conserv 35: 14-25.
81. Kirkby CA, et al. (2011) Closing the tourism-conservation loop in the Amazon. Environ Conserv 38 (1): 6–17.
82. Bowman M (2001) Economic Benefits of Nature tourism: Algonquin Park as a Case Study. M.A. Thesis, University of Waterloo, Ontario.
83. Bowman ME, Eagles PFG (2002) paper presented at the Parks Research Forum on Ontario, Ridgetown, Ontario, 25 April 2002.
84. Canadian Parks Service (1992) Economic Impact Analysis of Canals, National Historic Sites and National Parks in Ontario, Vol. II. Ottawa: Canadian Parks Service.
85. Pacific Analytics (2004) Economic Value of the Commercial Nature-Based Tourism Industry in British Columbia. Vancouver, Tourism British Columbia. 19 p.
86. Hunt LM, Englin J, Haider W (2005) Remote tourism and forest management: a spatial hedonc analysis. Ecol Econ 53: 101-113.
87. Stynes DJ (2006) National Park Visitor Spending and Payroll Impacts, 2006*.* East Lansing: Michigan State University.16 p.
88. Sun Y-Y, Stynes DJ, Propst DB (2002) Economic Impacts of Visitors to Mount Rainier National Park, 2000. East Lansing: Michigan State University. 16 p.
89. Moran D (1994) Contingent valuation and biodiversity: measuring the user surplus of Kenyan protected areas. Biodivers Conserv 3: 663-684.
90. Brown G, Swanson T, Moran D (1994) Optimally Pricing Games Reserves in Kenya. London: CSERGE.
91. Navrud S, Mungatana ED (1994) Environmental valuation in developing countries: the recreational value of wildlife viewing. Ecol Econ 11: 135-151.
92. Maille P, Mendelsohn R (1993) Valuing ecotourism in Madagascar. J Environ Manage 38: 213-218.
93. Kramer RA, Sharma N, Munasinghe M (1995) Valuing Tropical Forests: Methodology and Case Study of Madagascar. Washington, DC: World Bank. 65 p.
94. Mercer E, Kramer R, Sharma N (1995) Rain forest tourism – estimating the benefits of tourism development in a new national park in Madagascar. J Forest Econ 1: 239-269.
95. Clark C, Davenport L, Mkanga P (1995) Designing Policies for Setting User Fees and Allocating Proceeds among Stakeholders: the Case of Tarangire National Park, Tanzania. Washington, DC: World Bank.
96. Wells MP (1997) Economic Perspectives on Nature Tourism, Conservation and Development. Washington, DC: World Bank. 54 p.
97. Brown G, Ward M, Jansen DJ (1995) Economic Value of National Parks in Zimbabwe: Hwange and Mana Pools*.* Washington, DC: World Bank. 55 p.
98. Walpole MJ, Goodwin HJ, Ward KGR (2001) Pricing policy for tourism in protected areas: lessons from Komodo National Park, Indonesia. Conserv Biol 15: 218-227.
99. Firoozan AM, Abed MH, Bahmanpour H, Heshemi SA (2012) Estimated recreational value of Lahijan Forest using by contingent valuation method. J Agr Biol Sci 7: 659-663.
100. Lee C-K, Han S-Y (2002) Estimating the use and preservation values of national parks’ tourism resources using a contingent valuation model. Tourism Manage 23: 531-540.
101. Lee C-K (1997) Valuation of nature-based tourism resources using dichotomous choice contingent valuation method. Tourism Manage 18: 587-591.
102. Kaosa-Ard M, et al. (1995) Green Finance: Valuation and Financing of Kaho Yai National Par in Thailand. Bangkok: Thailand Development Research Institute. 4 p.
103. Kubíčková S, Grega L (2002) Willingness to pay for rural landscape preservation. In: Arberger A, Brandenburg C, Muhar A, editors. Monitoring and Management of Visitor Flows in Recreational and Protected Areas. Vienna: Bodenkultur University. pp. 335–339.
104. Gios G, Goio I, Notaro S, Raffaelli R (2006) The value of natural resources for tourism: a case study of the Italian Alps. Int J Tourism Res 8: 77-85.
105. Chase LC, Lee DR, Schulze WD, Anderson DJ (1998) Ecotourism demand and differential pricing of national park access in Costa Rica. Land Econ 74: 466-482.
106. Tobias D, Mendelsohn R (1991) Valuing ecotourism in a tropical rain-forest reserve. Ambio 20: 91-93.
107. Menkhaus S, Lober DJ (1996) International ecotourism and the valuation of tropical rainforests. J Environ Manage 47: 1-10.
108. Echeverría J, Hanrahan M, Solórzano R (1995) Valuation of non-priced amenities provided by the biological resources within the Monteverde Cloud Forest Preserve, Costa Rica. Ecol Econ 13: 43-52.
109. Loomis JB (2000) Economic values of wilderness recreation and passive use: what we think we know at the beginning of the 21^st^ century. USDA Forest Serv Proc RMRS-P-15 2: 5-13.
110. Shrestha RK, Loomis JB (2003) Meta-analytic benefit transfer of outdoor recreation economic values: testing out-of-sample convergent validity. Environ Resour Econ 25: 79-100.
111. Shrestha RK, Stein TV, Clark J (2007) Valuing nature-based recreation in the public natural areas of the Apalachicola River Region, Florida. J Environ Manage 85: 977-985.
